# A Novel Therapeutic Strategy for Bone Marrow Failure: Niche Rejuvenation Using Costal Cartilage‐Derived Stem Cells

**DOI:** 10.1002/advs.202507794

**Published:** 2025-08-27

**Authors:** Rui Dong, Zhiguo Ling, Pengyuan Fan, Debao Li, Jinsong Wang, Wenjiong Shi, Rui Zuo, Runfeng Chen, Xuemin Sun, Lang Xiao, Yushi Ran, Shucheng Huang, Yi Tian, Chao Zhang, Yuzhang Wu, Bing Ni, Yi Zhang

**Affiliations:** ^1^ Department of Pathophysiology College of High Altitude Military Medicine Army Medical University Chongqing 400038 China; ^2^ Institute of Immunology Army Medical University Chongqing 400038 China; ^3^ Chongqing International Institute for Immunology Chongqing 401338 China; ^4^ School of Pharmacy and Bioengineering Chongqing University of Technology Chongqing 400054 China; ^5^ Department of Orthopedics Xinqiao Hospital Army Medical University Chongqing 400038 China

**Keywords:** aplastic anemia, bone marrow niche, bone marrow stromal cells, costal cartilage‐derived stem cells, hematopoietic stem cells

## Abstract

The bone marrow (BM) niche plays a critical role in maintaining hematopoietic stem cell function but is highly vulnerable to damage from chemotherapy and radiation. However, current therapeutic strategies for BM niche failure remain significantly limited. The previous study demonstrate that costal cartilage‐derived stem cells (CDSCs) exhibit substantial self‐renewal and bone‐forming capacity; however, whether and how CDSCs contribute to BM microenvironment maintenance remains unknown. In this study, the co‐transplantation of CDSCs with multipotent progenitors (MPPs) successfully rescued lethally irradiated mice. By contrast, transplantation of mesenchymal stem cells with MPPs or MPPs alone fails to rescue the mice, suggesting a potential role of CDSCs in hematopoietic reconstitution. RNA‐seq and experimental data suggest that CDSCs are involved in rejuvenating the BM niche. Mechanistically, CDSCs not only differentiate into niche components, including bone marrow stromal cells, endothelial cells, and osteoblasts, but also secrete pro‐hematopoietic cytokines, thereby rejuvenating the irradiated microenvironment. Additionally, CDSCs protect residual hematopoietic stem and progenitor cells from radiation‐induced apoptosis and DNA damage while enhancing niche repair. Finally, through synergy with cyclosporine A, CDSCs markedly enhance hematopoietic recovery in mice with aplastic anemia. Collectively, these findings establish CDSCs as a versatile platform for treating BM failure via microenvironmental restoration.

## Graphical abstract

CDSCs exhibit the potential to differentiate into endothelial cells, vascular pericytes, and osteoblasts. These differentiated cells are capable of secreting additional pro‐hematopoietic cytokines. Furthermore, CDSCs can inhibit apoptosis and reduce DNA damage in injured hematopoietic stem and progenitor cells (HSPCs, including RHSCs and RHPCs), while enhancing the hematopoietic function of donor‐derived HSPCs, including DHSCs and DHSPCs.

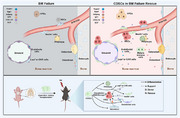

## 1 Introduction

The bone marrow (BM) niche is a complex microenvironment essential for maintaining hematopoietic stem cells (HSCs) and sustaining lifelong blood cell production.^[^ ^1^ ^]^ Comprised of both hematopoietic and supporting stromal cells, including perivascular cells, endothelial cells, osteoblasts, and mesenchymal stem cells (MSCs), the BM niche precisely regulates HSCs self‐renewal, differentiation, and stress responses through intricate cellular interactions.^[^ ^2^, ^3^ ^]^ However, this delicate system is particularly susceptible to damage from chemotherapy, radiation, immune dysregulation, as well as age‐related degeneration, often leading to severe hematopoietic impairment.^[^ ^4^ ^]^

While hematopoietic stem cell transplantation (HSCT) remains a cornerstone therapy for hematologic malignancies,^[^ ^5^ ^]^ its effectiveness is limited by two critical factors: the scarcity of donor HSCs^[^ ^1^ ^]^ and the frequent failure to address underlying niche damage,^[^ ^6^ ^]^ especially in non‐hematopoietic cell‐mediated pathologies. This has driven growing interest in developing therapies that directly target and repair the injured BM microenvironment.

The BM niche originates through endochondral ossification, where cartilage is progressively replaced by bone‐forming cells and vasculature.^[^ ^7^ ^]^ This developmental process depends on mesoderm‐derived stem cells, particularly MSCs and skeletal stem cells (SSCs),^[^ ^8^ ^]^ which naturally contribute to niche formation and maintenance. And preconditioning regimens involving irradiation or chemotherapy, though necessary for HSCT, often cause irreversible damage to the BM niche.^[^ ^9^, ^10^, ^11^ ^]^ Building on this biology, researchers have explored co‐transplanting bone marrow stromal cells (BMSCs) or MSCs with HSCs to improve transplantation outcomes.^[^ ^9^, ^12^ ^]^ While such approaches have shown some success in enhancing engraftment or reducing complications like graft‐versus‐host disease (GVHD), they remain constrained by the need for large numbers of hematopoietic stem progenitor cells (HSPCs) and demonstrate limited niche repair capacity.^[^ ^9^, ^12^ ^]^

Our previous work identified costal cartilage‐derived stem cells (CDSCs) as a novel cell population with remarkable regenerative potential.^[^ ^13^ ^]^ In rat models, CDSCs demonstrated superior self‐renewal and bone‐forming capacity compared to conventional BMSCs. However, whether and how the neonatal mouse CDSCs contribute to BM microenvironment maintenance remained unexplored. In this study, we demonstrated that CDSCs combined with multipotent progenitors (MPPs) effectively rescued lethally irradiated mice, whereas neither MPPs alone nor those co‐transplanted with MSCs exhibited therapeutic efficacy. Furthermore, CDSCs combined with minimal HSPCs transplantation achieved comparable hematopoietic reconstitution to BM transplantation (BMT). Mechanistically, CDSCs restored the irradiated BM niche through differentiating into endothelial cells, BMSCs and osteoblast and secreting pro‐hematopoietic growth factors. Additionally, CDSCs suppressed irradiation‐induced apoptosis in lineage‐negative (Lin^−^) cells and attenuated DNA damage accumulation in MPPs. In an aplastic anemia mouse model, CDSCs synergized with cyclosporine A (CsA) to significantly improve survival outcomes. These findings support CDSCs as a powerful strategy for BM niche regeneration to improve HSCT efficacy.

## 2 Results

### 2.1 Enzyme‐Isolated CDSCs Rescue BM Hematopoietic Failure Efficiently as BMT

**Figure 1:**
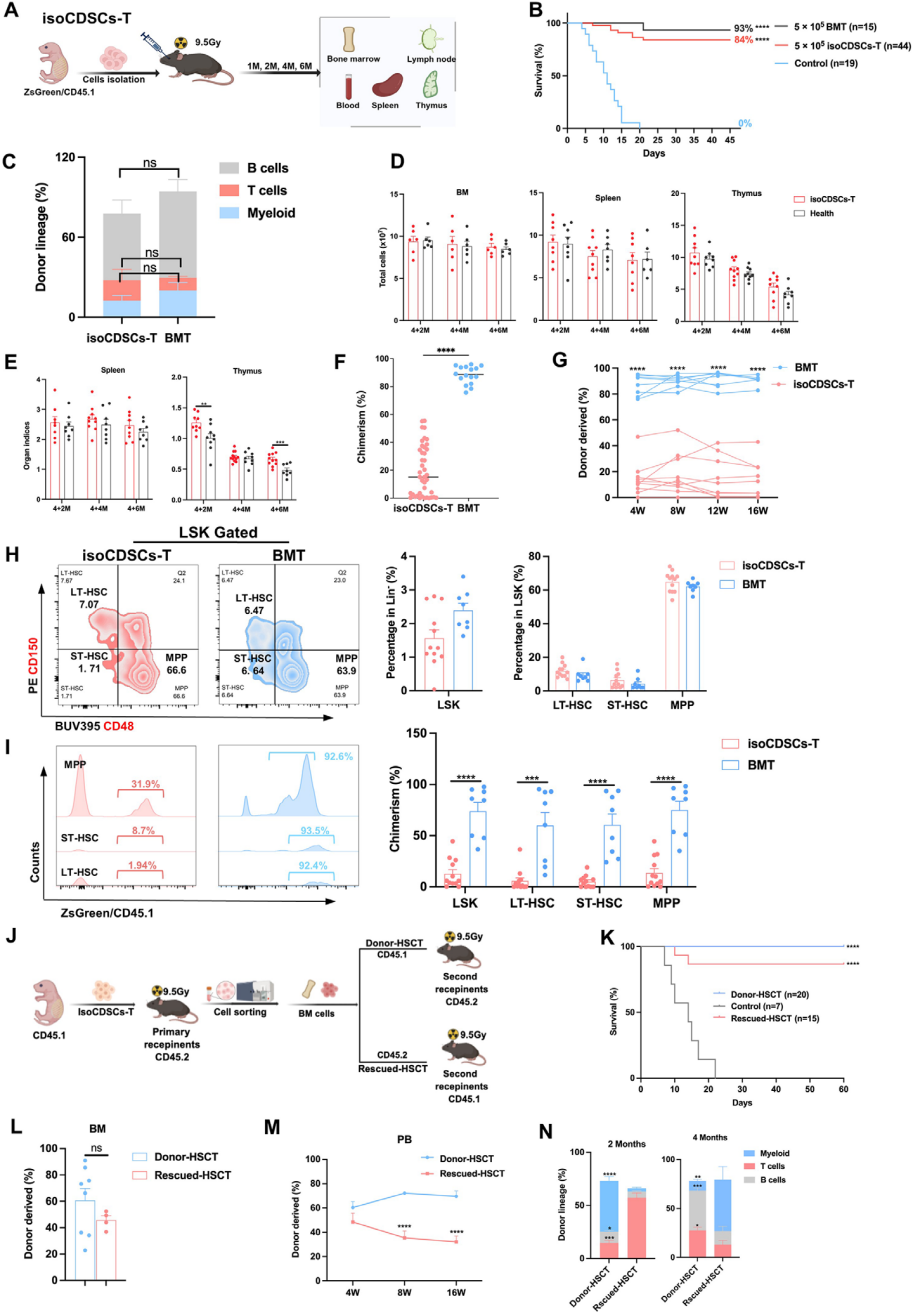
IsoCDSCs rescue lethally irradiated mice and achieve hematopoietic recovery comparable to that of BMT. A) Flowchart illustrating the process of isoCDSCs‐T generation. B) Survival curve of mice following transplantation with isoCDSCs‐T and bone marrow transplantation (BMT). C) Lineage distribution of donor‐derived cells (CD45.1/ZsGreen) in peripheral blood (PB) for isoCDSCs‐T (*n* = 7) and BMT (*n* = 6) at 8 weeks post‐transplantation. D,E) Comparison of total cell counts in BM (harvested from bilateral femurs and tibias), spleen, and thymus, and organ indices of the spleen and thymus between recipient mice (isoCDSCs‐T, *n* = 8) and health group (*n* = 8) at 16 weeks post‐transplantation. F) Comparison of chimerism levels in PB at 8 weeks post‐transplantation between isoCDSCs‐T (*n* = 41) and BMT (*n* = 16). G) Comparison of chimerism levels in PB at the indicated time points post‐transplantation between isoCDSCs‐T (*n* = 12) and BMT (*n* = 8). H,I) Percentage and donor chimerism of HSPCs in BM at 16 weeks post‐transplantation for isoCDSCs‐T (*n* = 12) and BMT (*n* = 8). J) Overview of non‐competitive transplantation of donor‐derived BM cells (Donor‐HSCT/CD45.1) and rescue of irradiated BM cells (Rescued‐HSCT/CD45.2) from primary recipients of isoCDSCs‐T at 16 weeks post‐transplantation. K) Survival rates of non‐competitive transplanted recipients of Donor‐HSCT and Rescued‐HSCT. L) Donor chimerism in BM for non‐competitive secondary transplanted recipients of Donor‐HSCT (*n* = 8) and Rescued‐HSCT (*n* = 4) at 16 weeks post‐transplantation. M) Donor chimerism in PB of non‐competitive transplanted recipients of Donor‐HSCT (*n* = 4) and Rescued‐HSCT (*n* = 5) at the indicated time points post‐transplantation. N) Lineage distribution of donor‐derived cells (CD45.1/CD45.2) in PB of non‐competitive secondary transplanted recipients: Donor‐HSCT (*n* = 4 at 2 months; *n* = 8 at 4 months) versus Rescued‐HSCT (*n* = 4 at 2 months; *n* = 4 at 4 months). Data presented as Mean ± SEM. ^*^ *p* < 0.05, ^**^ *p* < 0.01, ^***^ *p* < 0.001, ^****^ *p* < 0.0001, ns, not significant. P values were calculated by the Log‐rank test or two‐tailed Student's *t*‐test.

We previously reported that, as a novel cell population, CDSCs exhibit superior self‐renewal and bone‐forming capacity compared to BMSCs.^[^ ^13^ ^]^ However, whether CDSCs contribute to the establishment of an injured BM niche remains unclear. In this study, we enzymatically digested the costal cartilage of neonatal mice and harvested the resulting cells, which were termed enzyme‐isolated CDSCs (isoCDSCs). Subsequently, isoCDSCs were transplanted into lethally irradiated mice as illustrated in **Figure** **1**A. Notably, transplantation of 5 × 10^5^ isoCDSCs (isoCDSCs‐T) resulted in an 84% survival rate, comparable to that achieved with BMT (Figure 1B). Both isoCDSCs‐T and BMT demonstrated similar reconstitution of lymphoid and myeloid lineages in peripheral blood (Figure 1C). Furthermore, recipients of isoCDSCs‐T exhibited total cell counts and organ indices in BM, thymus, and spleen that were comparable to those of age‐matched healthy mice (Figure 1D,E), despite lower chimerism levels in peripheral blood compared to BMT (Figure 1F,G). Importantly, similar quantities and proportions of HSPCs were observed in both the isoCDSCs‐T and BMT groups (Figure 1H; Figure S1A,C,E, Supporting Information), although chimerism levels remained lower than those in BMT (Figure 1I; Figure S1B,D, Supporting Information). Consistently, analyses of mature blood cells in BM, T and B cell proportions in lymph nodes and thymus, and T cells, B cells, NK cells, and granulocytes in peripheral blood and spleen revealed no significant differences between isoCDSCs‐T and BMT (Figure S2A–L, Supporting Information).

To evaluate the long‐term multilineage reconstitution capacity, we conducted secondary non‐competitive transplantation experiments (Figure 1J). Both the rescued HSCT and the donor‐derived HSCT from isoCDSCs‐T recipients exhibited survival benefits (Figure 1K). Although the chimerism in peripheral blood was higher for donor‐derived HSCT, no significant difference was observed in BM (Figure 1L,M). The donor‐derived HSCT demonstrated classic multilineage reconstitution, whereas the rescued HSCT showed a T lymphoid‐biased reconstitution at two months and a myeloid‐biased reconstitution at four months post‐transplantation (Figure 1N). Furthermore, at 16 weeks after secondary transplantation, the rescued HSCT and the donor‐derived HSCT displayed comparable levels of chimerism and cell numbers, with the exception of granulocytes (Figure S3A–E, Supporting Information). Detailed flow cytometry clustering strategies are provided in Figure S4A–G (Supporting Information). Collectively, isoCDSCs‐T achieved hematopoietic reconstitution efficacy that is comparable to BMT, despite the significantly lower chimerism rate.

### 2.2 IsoCDSCs‐T Exhibit Superior Efficacy in Re‐Establishing the BM Niche Compared to BMT

**Figure 2:**
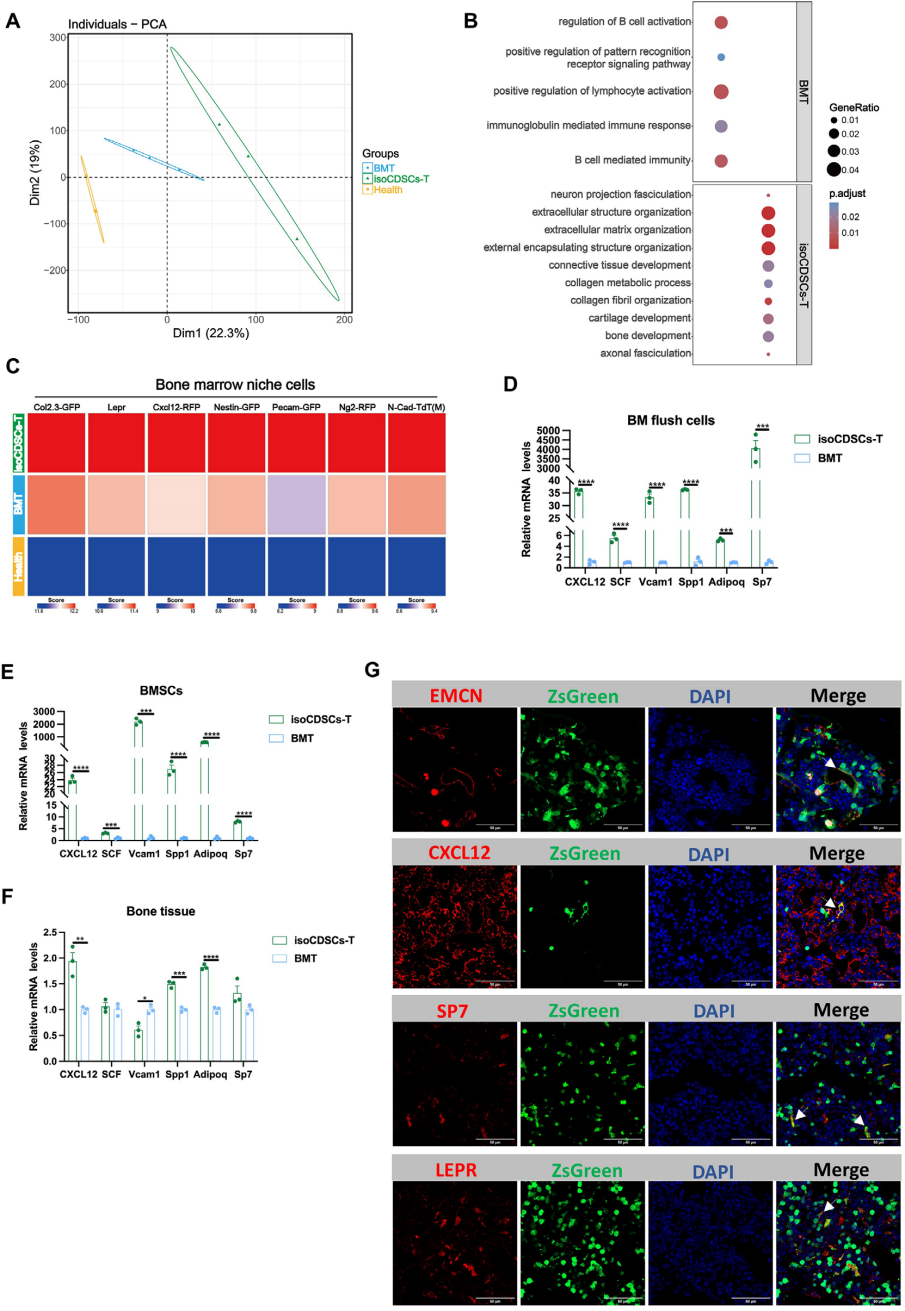
IsoCDSCs‐T exhibit the characteristic features of BMSCs. A) Principal component analysis (PCA) clustering of the healthy, BMT, and CDSCs transplantation groups. B) GO enrichment analysis of differentially expressed genes (DEGs) in the BMT and CDSCs transplantation groups. C) Abundance of infiltrating BM niche cells in the BMT and CDSCs transplantation groups. D–F) qRT‐PCR analysis of stroma‐derived factors in BM flush cells, BMSCs, and bone tissue at 2 weeks post‐transplantation in recipient mice from the BMT and CDSCs transplantation groups. G) Immunofluorescence imaging of BM tissues at 8 weeks post‐transplantation in recipient mice from the CDSCs transplantation group. Data presented as Mean ± SEM. ^*^ *p* < 0.05, ^**^ *p* < 0.01, ^***^ *p* < 0.001, ^****^ *p* < 0.0001. *p*‐values were calculated by the two‐tailed Student's *t*‐test.

To further investigate the mechanisms underlying the effects of isoCDSCs‐T that are comparable to those of BMT, we conducted RNA‐seq analysis on BM samples from BMT and isoCDSCs‐T recipients. PCA revealed distinct clustering of the three groups: healthy controls, BMT recipients, and isoCDSCs‐T recipients (**Figure** **2**A). Notably, GO term enrichment analysis demonstrated that genes highly enriched in the isoCDSCs‐T group were associated with extracellular structure organization, matrix remodeling, collagen metabolic processes, and cartilage and bone development processes, primarily linked to BM niche reconstruction. In contrast, the genes highly enriched in the BMT group were predominantly involved in the regulation of B‐cell and lymphocyte activation, as well as B‐cell‐mediated immunity (Figure 2B). Collectively, these GO analyses suggest that the isoCDSCs‐T group may exhibit a greater propensity for repairing the irradiation‐damaged BM niche compared to the BMT group.

Subsequently, based on a previous study,^[^ ^14^ ^]^ we examined the correlation between the gene profiles of our three groups and the top 800 genes (Table S2, Supporting Information) associated with various BMSCs, osteoblasts, and vascular endothelial cells. Our analysis revealed that the isoCDSCs‐T group exhibited a higher correlation with these BM niche‐related cell types compared to the other two groups (Figure 2C). Additionally, the isoCDSCs‐T group demonstrated significantly higher expression levels of multiple pro‐hematopoietic factors, such as CXCL12, Kitl (SCF), Vcam1, Spp1, Adipoq, and Sp7, which have been previously reported to promote hematopoiesis^[^ ^9^ ^]^ (Figure 2D–F). Immunofluorescence staining further confirmed that isoCDSCs‐T expressed markers characteristic of endothelial cells (endomucin), perivascular cells (CXCL12 and Lepr), and osteoblasts (Sp7) (Figure 2G). However, BMT‐derived cells exhibited minimal co‐expression of BMSC markers (Figure S5, Supporting Information). Collectively, these findings suggest that isoCDSCs‐T may be involved in hematopoietic reconstitution by facilitating the re‐establishment of the irradiation‐damaged BM niche.

### 2.3 CDSCs Co‐transplantation Promotes Hematopoietic Reconstitution through Enhancement of the Bone Marrow Niche

**Figure 3:**
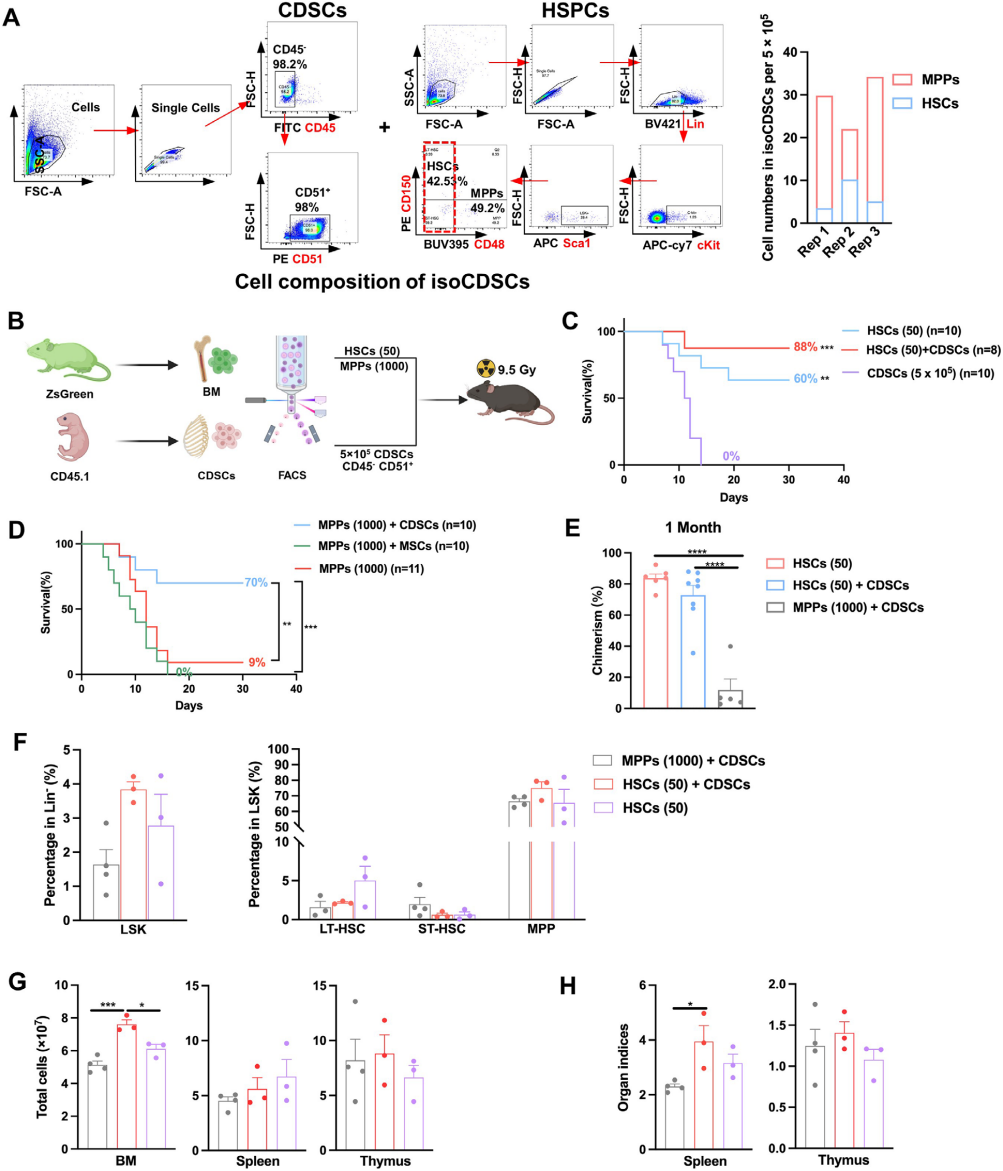
Co‐transplantation of CDSCs with HSPCs successfully rescues lethally irradiated mice. A) Compositional analysis of isoCDSCs revealed a predominant population of CD45^−^ CD51^+^ CDSCs (left panel) and minor fractions of HSCs/MPPs (middle panel). Quantification showed HSC/MPP numbers per transplanted mouse (5 × 10^5^ cells). B) Overview of HSCs and MPPs transplanted either alone or in combination with CDSCs. C) Survival rates following transplantation of HSCs and CDSCs, either alone or co‐transplanted. Cells were transplanted into lethally irradiated mice via intraorbital vein injection. D) Survival rates following transplantation of MPPs either alone or in combination with CDSCs or MSCs. E) Donor chimerism levels in PB at 4 weeks post‐transplantation for HSCs alone (*n* = 6), HSCs + CDSCs (*n* = 8), and MPPs + CDSCs (*n* = 5). F) Percentage of HSPCs in BM at 8 weeks post‐transplantation for HSCs alone (*n* = 3), HSCs + CDSCs (*n* = 3), and MPPs + CDSCs (*n* = 4). G,H) Analysis of total cell counts in BM (harvested from bilateral femurs and tibias), spleen, and thymus, and organ indices of the spleen and thymus at 8 weeks post‐transplantation for HSCs and MPPs transplanted either alone or in combination with CDSCs. Data presented as Mean ± SEM. ^*^ *p* < 0.05, ^**^ *p* < 0.01, ^***^ *p* < 0.001, ^****^ *p* < 0.0001. *p*‐values were calculated by the Log‐rank test or one‐way ANOVA test.

To investigate the cellular mechanisms underlying the effects of isoCDSCs‐T on hematopoietic reconstitution, we conducted a comprehensive analysis of the cellular composition of isoCDSCs. Flow cytometric analysis demonstrated that the isoCDSCs population was predominantly composed of CD45^−^CD51^+^ cells (namely CDSCs), accounting for ≈98%, with only a minimal presence of HSPCs, including HSCs and MPPs (**Figure** **3**A). Specifically, among 5 × 10^5^ isoCDSCs, only 5.65 HSCs and 24.35 MPPs were identified (Figure 3A). To evaluate the potential of CDSCs to rejuvenate the BM niche during hematopoietic reconstitution following irradiation injury, we transplanted 5 × 10^5^ FACS‐sorted CDSCs in combination with either 50 purified HSCs or 1000 MPPs into lethally irradiated mice (Figure 3B). The results demonstrated that neither CDSCs nor MPPs alone could rescue lethally irradiated mice, whereas the specified number of HSCs alone rescued 60% of irradiated mice (Figure 3C,D). Notably, the combination of CDSCs with HSCs rescued 88% of irradiated mice (Figure 3C). Interestingly, co‐transplantation of CDSCs and MPPs also rescued 70% of irradiated mice (Figure 3D), suggesting that CDSCs may possess the capacity to rejuvenate the damaged BM niche, thereby providing an optimal hematopoietic microenvironment for MPPs. This is consistent with the established knowledge that MPPs lack long‐term hematopoietic reconstitution potential.^[^ ^15^ ^]^ Given that MSCs have been reported to facilitate the recovery of the damaged BM niche,^[^ ^15^ ^]^ we further examined the co‐transplantation of MSCs and MPPs. However, this approach failed to rescue any experimental mice (Figure 3D), indicating that CDSCs exhibit superior BM niche maintenance capabilities compared to MSCs.

Peripheral blood chimerism analysis demonstrated that transplantation of CDSCs + MPPs resulted in a similarly low level of chimerism as observed in the isoCDSCs‐T group (Figure 3E). Furthermore, the analysis of HSPC proportions revealed that CDSCs + MPPs exhibit hematopoietic reconstitution efficacy comparable to that of HSCs (Figure 3F). Similarly, CDSCs + MPPs showed consistency with HSCs in terms of organ indices and total cell counts in the BM, thymus, and spleen (Figure 3G,H). Collectively, these findings suggest that CDSCs likely facilitate hematopoietic reconstitution by enhancing the bone marrow microenvironment.

### 2.4 CDSCs Rejuvenate the Damaged BM Niche to Facilitate Hematopoiesis

**Figure 4:**
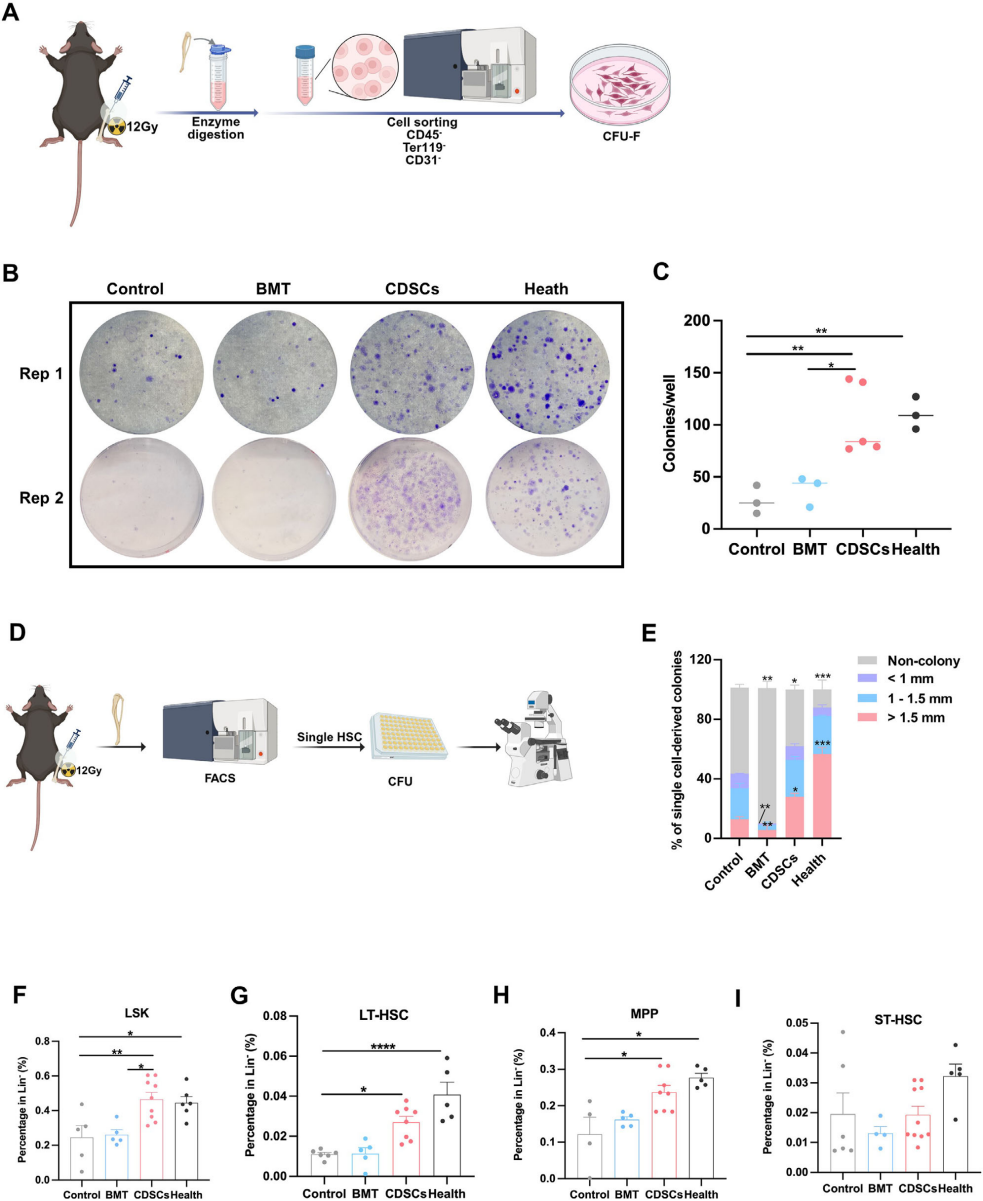
CDSCs rejuvenate the damaged BM niche to enhance hematopoietic recovery. A) Overview of the sorting of BMSCs from irradiated tibiae at 3 weeks post‐transplantation. B,C) Colony‐forming unit fibroblast (CFU‐F) assays in BMSCs for CDSCs, BMT, control, and healthy groups at 3–6 weeks post‐transplantation. D) Overview of the sorting of single HSCs from CDSCs, BMT (2‐3 weeks post‐transplantation), control, and health groups. E) Colony size of 100 sorted single HSCs after 14 days of methylcellulose culture. F–I) Percentage of HSPCs, including LSKs, LT‐HSCs, MPPs, and ST‐HSCs, in the CDSCs (*n* = 8), BMT (*n* = 5), control (*n* = 5), and health (*n* = 6) groups. Data presented as Mean ± SEM. ^*^ *p* < 0.05, ^**^ *p* < 0.01, ^***^ *p* < 0.001, ^****^ *p* < 0.0001. P values were calculated by the one‐way ANOVA test.

The irradiation permanently damages the BM stromal niche, which is difficult to repair through circulation.^[^ ^9^ ^]^ To investigate the effects of CDSCs on the irradiated BM niche and the restoration of hematopoietic function, we performed colony‐forming unit fibroblast (CFU‐F) assays and demonstrated that CDSCs transplantation significantly enhanced total BMSCs proliferation compared to controls and BMT (**Figure** **4**A–C). This enhanced CFU‐F activity promoted HSC function, as evidenced by increased large clone formation and reduced failed clones in single HSC CFU assays (Figure 4D,E). Additionally, CDSCs increased the percentage and number of Lin^−^ Sca1^+^ cKit^+^ cells (LSKs) and long‐term hematopoietic stem cells (LT‐HSCs) compared to controls (Figure 4F,G; Figure S6A,B, Supporting Information), as well as the percentage of MPPs relative to controls (Figure 4H). However, no significant differences were observed in the percentage and number of short‐term hematopoietic stem cells (ST‐HSCs) or the number of MPPs (Figure 4I; Figure S6C,D, Supporting Information). Collectively, these findings suggest that CDSCs play a critical role in rejuvenating the irradiated BM niche, thereby promoting hematopoietic reconstitution.

### 2.5 CDSCs Regenerated BM Endothelial and Stromal Cells in the Impaired BM Niche

**Figure 5:**
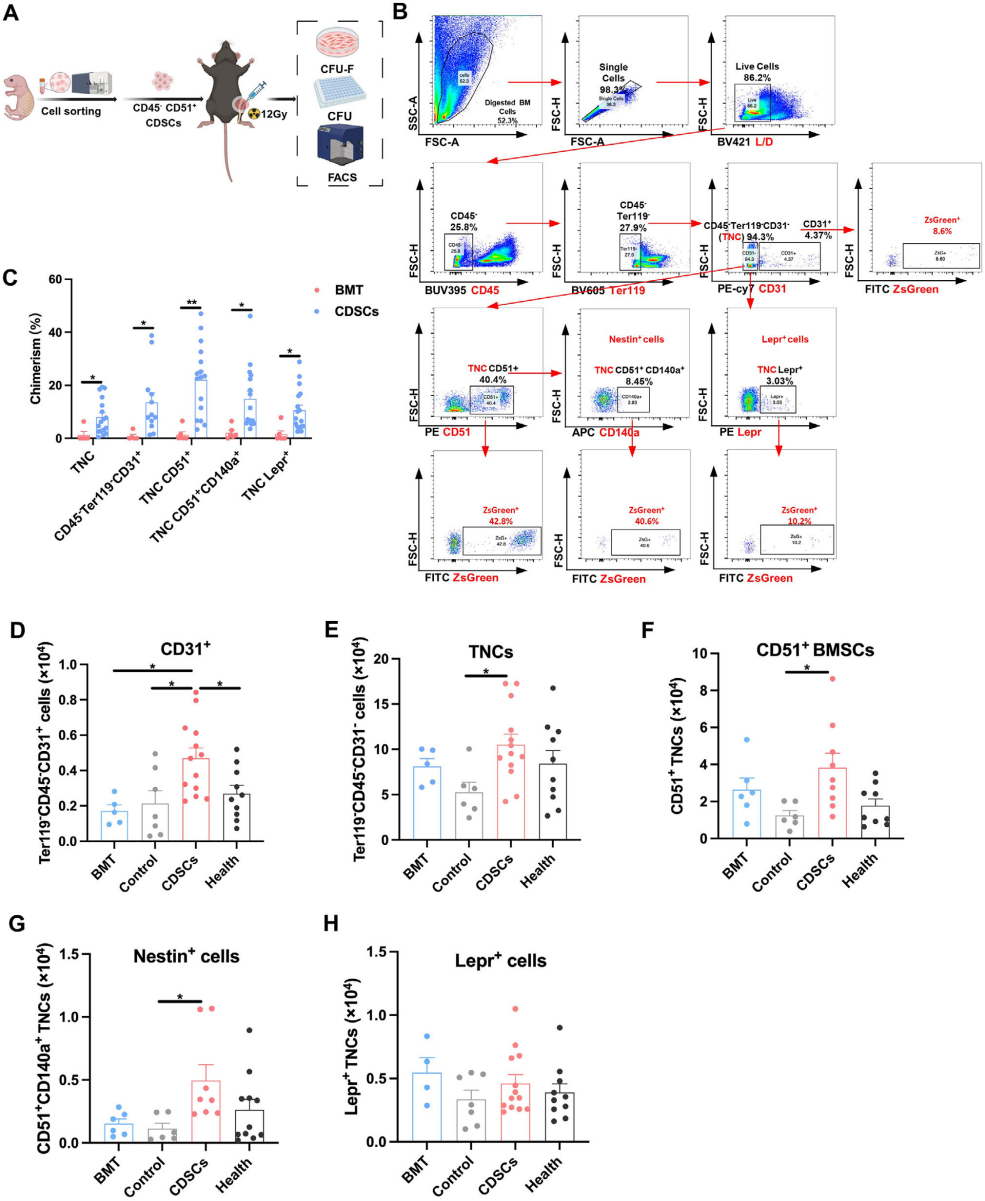
CDSCs differentiate into multiple types of BM niche cells within the irradiated BM niche. A) Summary of the transplantation of purified CDSCs and BM into the irradiated mouse tibiae. B) Flow cytometry plots illustrating BMSCs differentiated from CDSCs. C) Donor chimerism in BMSCs of irradiated tibiae following transplantation of purified CDSCs (*n* = 17) and BMT (*n* = 6) within 3–6 weeks post‐transplantation. D–H) Quantification of differentiated BMSCs in irradiated tibiae for the purified CDSCs (*n* = 13), BMT (*n* = 6), control (*n* = 7), and health (*n* = 11) groups. Data presented as Mean ± SEM. ^*^ *p* < 0.05, ^**^ *p* < 0.01. *p*‐values were calculated by the two‐tailed Student's *t*‐test or one‐way ANOVA test.

To further investigate the mechanisms by which CDSCs rejuvenate the irradiated BM niche, we established a mouse model involving localized irradiation of a single tibia followed by orthotopic injection of purified CDSCs and BM cells (**Figure** **5**A). After 3–6 weeks, flow cytometry analysis revealed that CDSCs differentiated into CD45^−^ Ter119^−^ CD31^−^ triple‐negative cells (namely total BMSCs), CD51^+^ BMSCs, endothelial cells, and perivascular cells (Nestin^+^ and Lepr^+^ cells) (Figure 5B). Compared to BMT, CDSCs exhibited enhanced differentiation into endothelial cells and BMSCs (Figure 5C). The morphology of BMSCs derived from CDSCs was consistent with that of healthy BMSCs (Figure S6E, Supporting Information), and they demonstrated a higher proliferation capacity than irradiated BMSCs in vitro (Figure S6F,G, Supporting Information). CDSCs transplantation significantly increased the number of vascular endothelial cells in the BM compared to BMT and control groups, even surpassing levels observed in healthy mice (Figure 5D). Additionally, the total numbers of BMSCs, CD51^+^ BMSCs, and Nestin^+^ BMSCs were significantly higher in CDSCs recipients than in controls (Figure 5E–G), although no significant difference was observed for Lepr^+^ BMSCs (Figure 5H). Collectively, these findings indicate that CDSCs can differentiate into endothelial cells and BMSCs within the damaged BM niche, thereby enhancing the recovery of irradiated endothelial cells and BMSCs.

### 2.6 Cultured CDSCs Exhibit BM Stromal Phenotype and Enhance Hematopoietic Recovery

**Figure 6:**
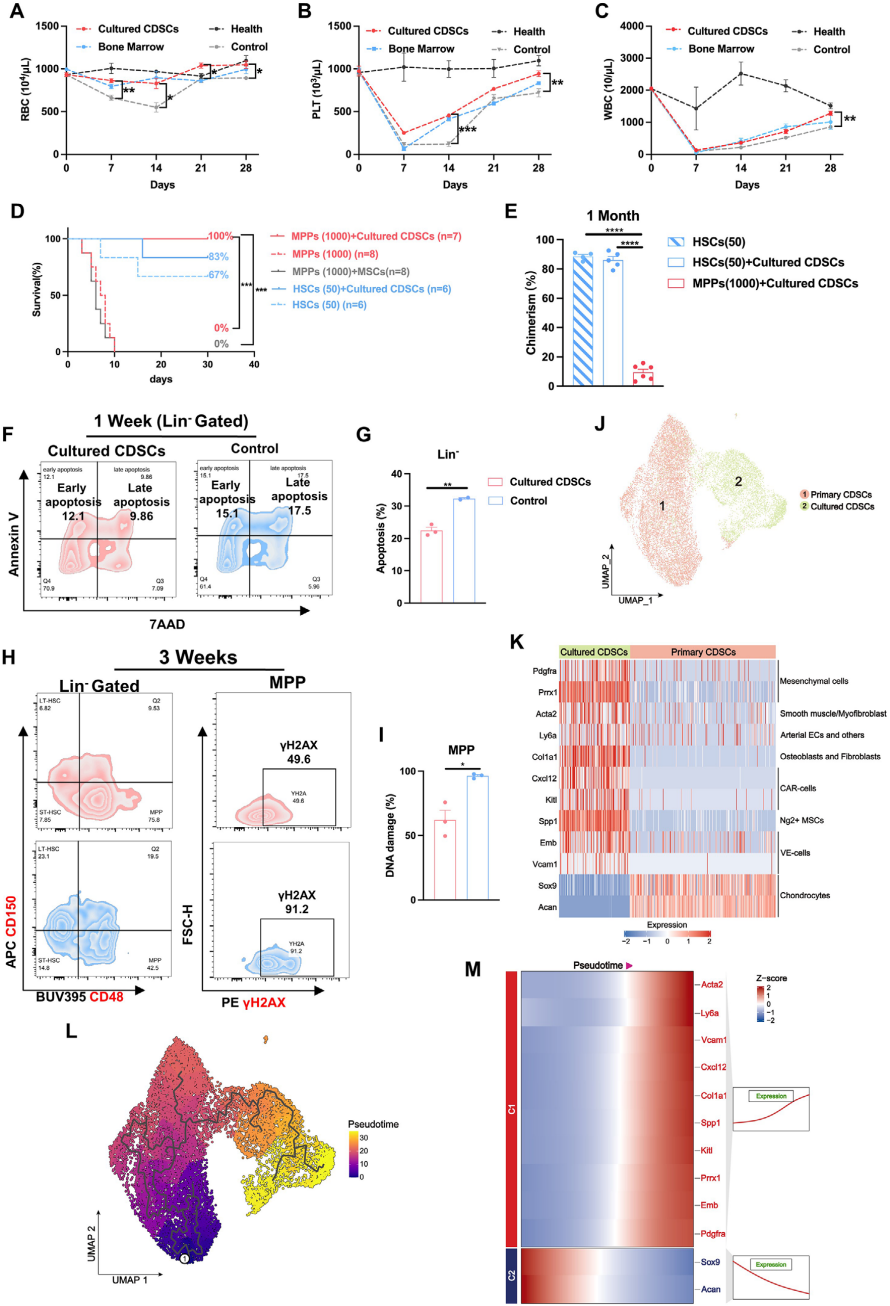
Cultured CDSCs exhibit the characteristic features of BMSCs and promote hematopoietic recovery. A–C) Hematological parameters (RBC, PLT, WBC) at 1–4 weeks post‐transplantation in the cultured CDSCs (*n* = 9), BMT (*n* = 5), control (*n* = 9), and healthy (*n* = 5) groups. D) Survival rates following transplantation of various amounts of HSCs (50) or MPPs (1000), either alone or co‐transplanted with cultured CDSCs (5 × 10^5^), as well as MPPs (1000) co‐transplanted with MSCs (5 × 10^5^), into lethally irradiated 8–12 weeks old mice. HSCs and MPPs were transplanted via intraorbital vein injection, while cultured CDSCs and MSCs were transplanted through bilateral intramedullary injection. E) Donor chimerism of HSCs (50) or MPPs (1000), either alone or co‐transplanted with cultured CDSCs, in the PB of recipient mice at 4 weeks post‐transplantation. Groups included: HSCs (50) alone (*n* = 4), HSCs (50) + cultured CDSCs (*n* = 5), and MPPs (1000) + cultured CDSCs (*n* = 7). F,G) Levels of apoptosis in BM Lin^−^ cells at 1 week post‐transplantation of cultured CDSCs. H,I) DNA damage levels in BM MPPs at 3 weeks post‐transplantation of cultured CDSCs. J) UMAP clustering analysis of primary and cultured CDSCs. K) Expression of genes associated with BMSCs, endothelial cells, and chondrocytes in primary and cultured CDSCs. L) Developmental and differentiation trajectories of primary and cultured CDSCs. M) Pseudotime trajectory analysis of genes related to BMSCs, endothelial cells, and chondrocytes between primary and cultivated CDSCs. Data presented as Mean ± SEM. ^*^ *p* < 0.05, ^**^ *p* < 0.01, ^***^ *p* < 0.001, ^****^ *p* < 0.0001. P values were calculated by the one‐way ANOVA test, the Log‐rank test or the two‐tailed Student's *t*‐test.

To evaluate the therapeutic potential of CDSCs, we cultured and expanded FCM‐sorted CDSCs in vitro and subsequently treated sub‐lethally irradiated mice with either the cultured CDSCs or BM cells (Figure S7A, Supporting Information). Results showed that the cultured CDSCs significantly accelerated recovery of red blood cells (RBCs), platelets (PLTs), and white blood cells (WBCs) in peripheral blood compared to controls (**Figure** **6**A–C). Notably, in the femoral transplantation experiment involving lethally irradiated mice, co‐transplantation of cultured CDSCs with MPPs effectively rescued the mice (Figure 6D). In contrast, mice in the group receiving co‐transplantation of MSCs and those in the group receiving MPPs alone did not survive (Figure 6D). For co‐transplantation with HSCs, the survival rates and chimerism levels were comparable to those of HSC alone (Figure 6D,E). The co‐transplantation results of primary and cultured CDSCs, respectively with HSCs or MPPs demonstrated that cultured CDSCs exhibited a comparable survival rate and chimerism level. Mechanistically, cultured CDSCs inhibited apoptosis of Lin^−^ BM cells one week after irradiation injury (Figure 6F,G) and suppressed DNA damage in cKit^+^ cells and MPPs at three weeks post‐irradiation (Figure 6H,I; Figure S7B, Supporting Information). Previous studies suggested that irradiation could trigger the senescence of HSC, and the higher expression of CD150 signified the senescence of LT‐HSC.^[^ ^16^, ^17^ ^]^ In our study, by four weeks, the cultured CDSCs inhibited the senescence of LT‐HSCs and maintained normal proportions, as well as increased MPPs, CMPs, and GMPs (Figure S7C,D, Supporting Information).

To determine whether there were any differences between the cultured CDSCs and the primary CDSCs, we performed single‐cell RNA sequencing on these two groups of cells. Based on the UMAP analysis, primary and cultured CDSCs were classified into two population of cells (Figure 6J). Analysis of gene expression in BMSCs, vascular endothelial cells, and chondrocytes between primary and cultured CDSCs indicated that cultured CDSCs highly expressed BMSC markers, such as CXCL12, Pdgfra, Prrx1, Kitl, Spp1, Acta2, as well as endothelial cells markers, such as Ly6a, Vcam1, and Emb, and osteoblast markers, such as Col1a1, but the expression of chondrocyte markers, such as Sox9 and Acan, was decreased (Figure 6K; Figure S8A, Supporting Information). Pseudotime trajectory revealed that the cultured CDSCs had the potential developmental trajectory of BM niche‐related genes, while the developmental trajectory of chondrocyte decreased (Figure 6L,M). Flow cytometry confirmed that primary CDSCs lacked expression of BMSC markers except for Sca1 (Figure S8B, Supporting Information). In contrast, the cultured CDSCs expressed several BMSC markers. Specifically, ≈50% of cultured CDSCs expressed markers of CXCL12‐abundant reticular (CAR) cells, 60% expressed Nestin+ cell markers, and 40% expressed Sca1, a marker of endothelial cells (Figure S8C, Supporting Information). The expression of BM stromal markers increased with passage number of CDSCs (Figure S8D, Supporting Information). These indicated that cultured CDSCs arise BM stromal characteristic accompanying the decreased of the feature of cartilage compared to primary CDSCs.

Collectively, cultured CDSCs not only repair the BM niche, but also inhibit the apoptosis and DNA damage of injury HSPCs, thereby facilitating hematopoietic recovery.

### 2.7 Cultured CDSCs Mitigate BM Failure in an Aplastic Anemia Model

**Figure 7:**
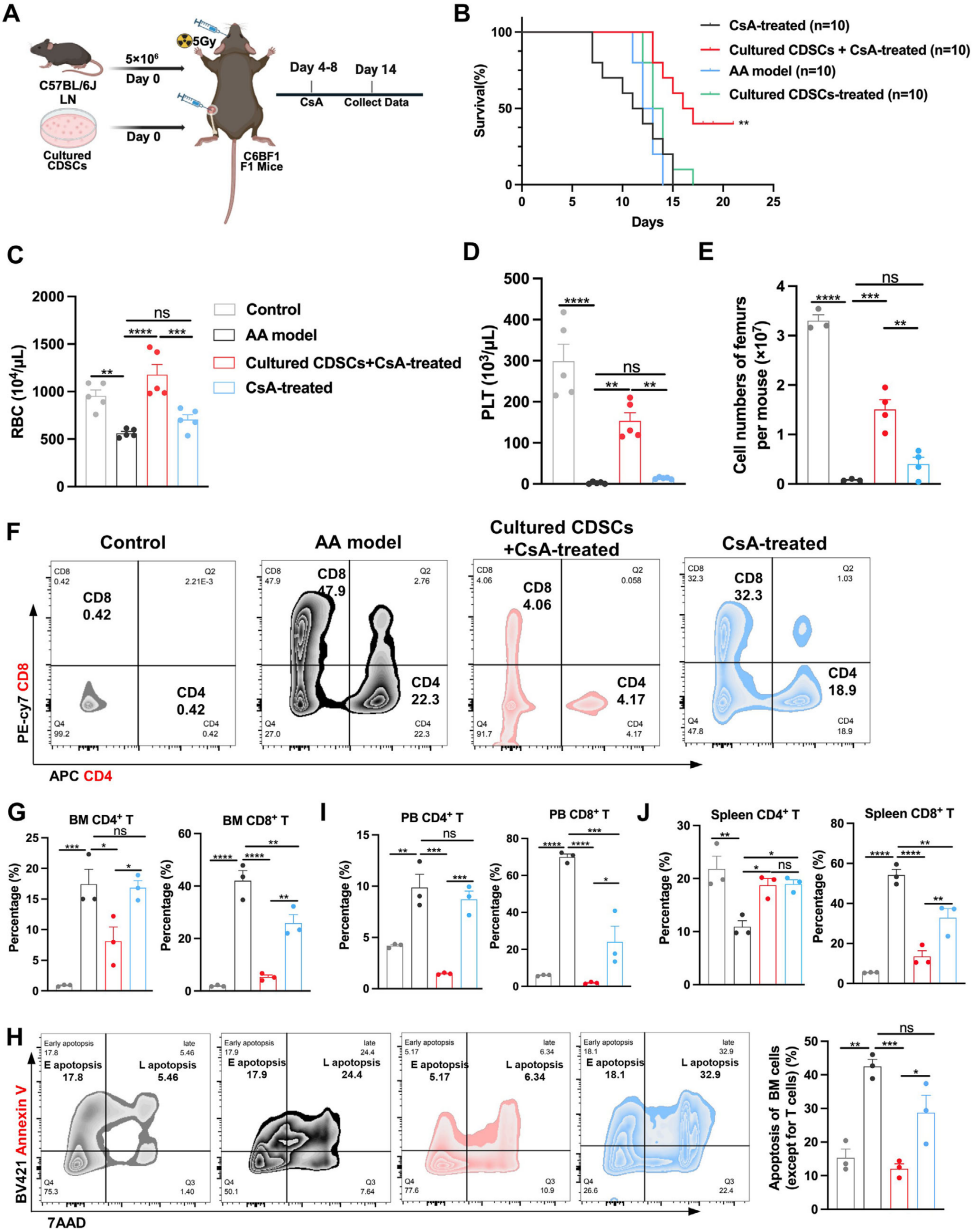
Cultured CDSCs in combination with CsA enhance survival in mice with aplastic anemia. A) Summary of the treatment using cultured CDSCs in combination with CsA in aplastic anemia (AA) mice. B) Survival rates in the AA model, control, CsA‐treated, and Cultured CDSCs + CsA‐treated groups. C–E) Hematological parameters (RBC, PLT) and total BM cell counts at 12–16 days post‐AA induction. Gray, black, red, and blue represent the Control group (*n* = 5), AA model group (*n* = 5), Cultured CDSCs + CsA‐treated group (*n* = 5), and CsA‐treated group (*n* = 5), respectively. F,G) Percentage of CD4^+^ and CD8^+^ T cells in BM for the AA model, control, CsA‐treated, and Cultured CDSCs + CsA‐treated groups at 12–16 days post‐transplantation. H) Flow cytometry plots and the percentage of apoptosis of BM cells (excluding CD4^+^ and CD8^+^ T cells) in the AA model (*n* = 3), control (*n* = 3), CsA‐treated (*n* = 3), and Cultured CDSCs + CsA‐treated groups (*n* = 3) at 12–16 days after the establishment of the AA mouse model. I,J) Percentage of CD4^+^ and CD8^+^ T cells in PB and spleen for the AA model, control, CsA‐treated, and Cultured CDSCs + CsA‐treated groups at 12–16 days post‐transplantation. Data presented as Mean ± SEM. ^*^ *p* < 0.05, ^**^ *p* < 0.01, ^***^ *p* < 0.001, ^****^ *p* < 0.0001, ns, not significant. *p*‐values were calculated by the Log‐rank test or the one‐way ANOVA test.

Aplastic anemia is a disease characterized by BM microenvironment failure, which manifests as pancytopenia and BM hypoplasia.^[^ ^18^ ^]^ Consequently, the cultured CDSCs may serve as a potential therapeutic option for aplastic anemia treatment. We established an aplastic anemia mouse model and treated mice with cultured CDSCs or primary CDSCs combined with CsA (**Figure** **7**A; Figure S9A, Supporting Information). While cultured or primary CDSCs alone slightly prolonged survival, combining them with CsA significantly improved survival compared to CsA alone (Figure 7B; Figure S9B, Supporting Information). Co‐treatment with CsA enhanced RBC and PLT recovery compared to CsA alone or untreated aplastic anemia mice (Figure 7C,D), though no significant differences were observed in WBCs, neutrophils (NEUT), or reticulocytes (RET) (Figure S9C–F, Supporting Information). Co‐treatment also increased total BM cells, LSKs, and MPPs (Figure 7E; Figure S9F,G, Supporting Information), inhibited BM CD4^+^ and CD8^+^ T cell infiltration (Figure 7F,G), and reduced BM cell apoptosis (except for BM CD4^+^ and CD8^+^ T cells) (Figure 7H). Similar effects were observed in the peripheral blood and spleen (Figure 7I,J). These results suggest that cultured CDSCs hold therapeutic potential for aplastic anemia.

## 3 Discussion

**Figure 8:**
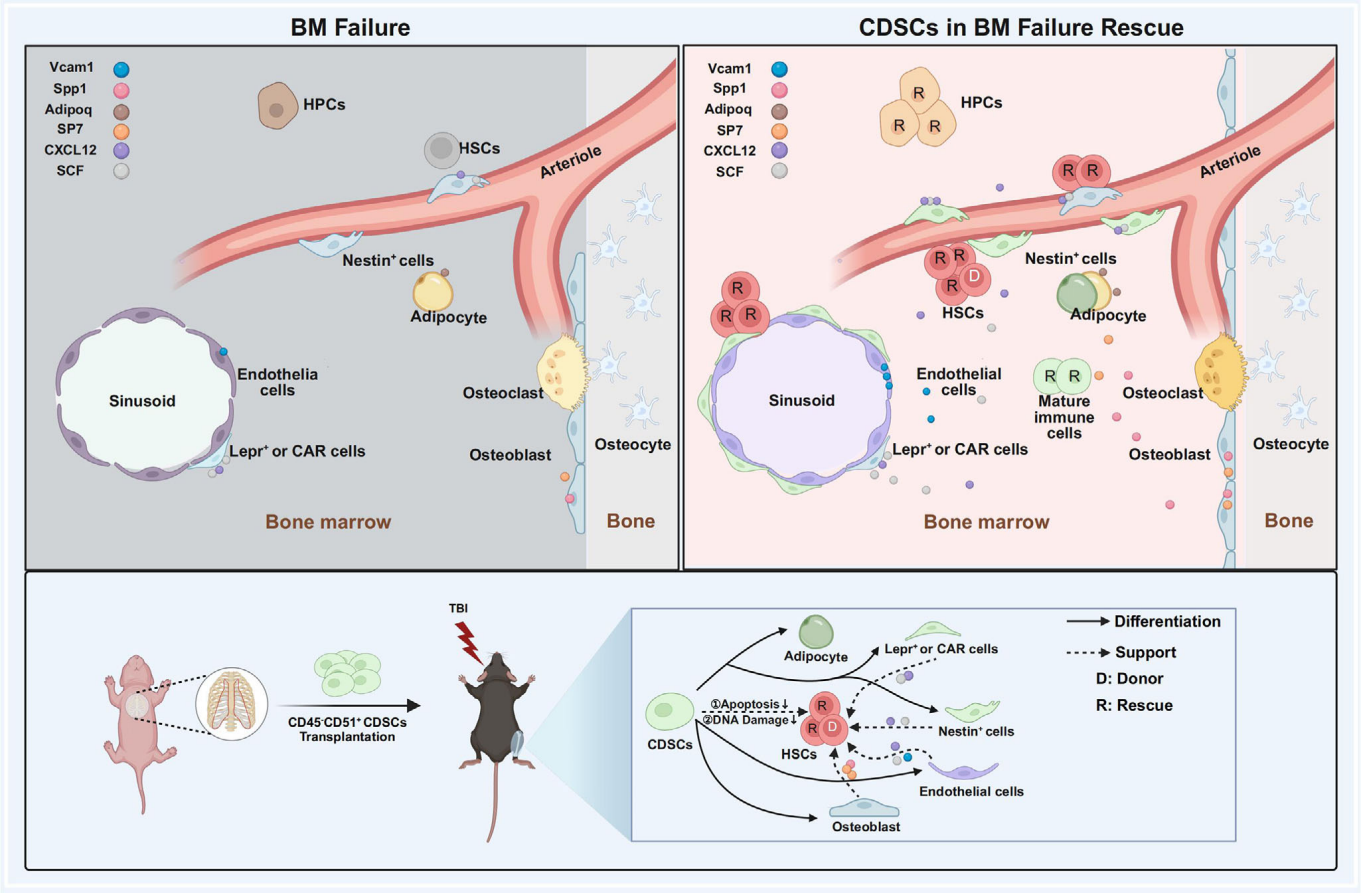
A schematic diagram illustrating the mechanism by which costal cartilage‐derived stem cells (CDSCs) regenerate damaged BM niche to facilitate hematopoietic reconstitution. BM failure involves damage to both hematopoietic and non‐hematopoietic cells within the BM microenvironment. CDSCs possess the potential to differentiate into endothelial cells, vascular pericytes (including Nestin^+^ cells, CAR cells, and Lepr^+^ cells), as well as osteoblasts. These differentiated cells are capable of secreting additional pro‐hematopoietic cytokines. Furthermore, CDSCs can inhibit apoptosis in injured HSPCs (including rescued HPCs and HSCs, referred to as RHPCs and RHSCs) and enhance their proliferation (including donor‐derived HSPCs, DHSPCs and RHSPCs). In summary, CDSCs effectively restore the function of BM failure.

Although CDSCs have predominantly been investigated for applications in skeletal tissue engineering,^[^ ^19^, ^20^ ^]^ in this study, we reveal their innovative potential to reconstruct the hematopoietic microenvironment (**Figure** **8**). CDSCs not only differentiated into essential niche components, such as perivascular cells, endothelial cells, and osteoblasts, but also secreted critical hematopoietic cytokines and safeguarded HSPCs from radiation‐induced damage. This dual mechanism of structural and functional niche restoration constitutes a substantial advancement in strategies for hematopoietic recovery. Furthermore, CDSCs hold considerable promise as a therapeutic approach for aplastic anemia.

Our findings demonstrate the exceptional capacity of CDSCs to facilitate hematopoietic reconstitution with minimal support from HSPCs. Although MPPs alone are incapable of sustaining long‐term engraftment due to their limited self‐renewal ability,^[^ ^21^ ^]^ their combination with CDSCs resulted in a significant improvement in survival rates. Conventionally, at least 5 × 10^5^ BM cells containing ≈300 HSCs are required for transplantation to ensure adequate hematopoietic reconstitution.^[^ ^22^, ^23^ ^]^ In stark contrast, our CDSCs‐based approach achieved comparable hematopoietic functionality with fewer than 10 HSCs, representing a dramatic reduction in the required number of HSPCs. A comprehensive analysis of hematopoietic progenitor populations, mature immune cell reconstitution, and organ recovery confirmed that CDSCs‐supported transplants performed equivalently to BMT.

We observed that the chimerism levels in our isoCDSCs‐T group were significantly lower than those in the BMT group. A previous study demonstrated that moderate to high doses of whole‐body radiation can impair the function of HSCs, leading to acute or prolonged myelosuppression.^[^ ^16^ ^]^ Despite administering a fully myeloablative radiation dose (9.5 Gy), which typically eradicates endogenous HSPCs, recipients of CDSCs maintained functional hematopoiesis with minimal contribution from donor cells. This suggests that CDSCs may protect residual host HSPCs from radiation‐induced damage, as supported by our findings of reduced apoptosis and DNA damage in Lin^−^ cells and MPPs.^[^ ^24^, ^25^, ^26^, ^27^ ^]^ Furthermore, these protected HSPCs retained their full functional potential, as evidenced by their capacity for long‐term engraftment in secondary transplants and competitive repopulation assays.

The BM niche plays a pivotal role in the maintenance and expansion of HSCs.^[^ ^28^ ^]^ However, the irradiation or chemotherapy administered prior to HSCT to eradicate host HSPCs also inflicts substantial damage on the BM niche, affecting both hematopoietic and non‐hematopoietic cells.^[^ ^9^, ^29^ ^]^ CDSCs exhibit remarkable plasticity, differentiating into multiple niche cell types, including BMSCs (Nestin^+^, CAR cells, and Lepr^+^), endothelial cells, and osteoblasts. These regenerated cells likely contribute to niche function through the production of essential factors such as CXCL12 and SCF.^[^ ^30^, ^31^, ^32^, ^33^, ^34^, ^35^ ^]^ Importantly, CDSCs enhance CFU‐F activity and increase the production of multiple pro‐hematopoietic cytokines, including CXCL12, Kitl, Vcam1, Spp1, Adipoq, and Sp7. Our findings demonstrate that CDSCs can effectively regenerate the damaged microenvironment via two key mechanisms: differentiation into functional niche cells and secretion of critical hematopoietic cytokines.

CDSCs demonstrated significantly enhanced niche‐restorative capacity compared to MSCs. MSCs are recognized for their ability to facilitate hematopoietic recovery by repairing the damaged BM niche and secreting pro‐hematopoietic cytokines.^[^ ^15^, ^36^ ^]^ In our study, we observed that co‐transplantation of CDSCs and MPPs markedly improved survival, whereas co‐transplantation of MSCs and MPPs failed to rescue the mice. These findings suggest that CDSCs may possess a more robust capacity for BM niche repair than MSCs. Additionally, a previous study revealed that co‐transplantation of CD73^+^CD105^−^Sca1^+^ BMSCs with HSCs can increase the number of HSCs and enhance B lymphopoiesis.^[^ ^9^, ^37^, ^38^, ^39^ ^]^ Similarly, co‐transplantation of endothelial cells with HSCs also promotes regenerative hematopoiesis.^[^ ^9^, ^37^, ^38^, ^39^ ^]^ However, the application of these cell types is limited due to their extremely low cell counts, difficulty in in vitro expansion, or loss of function. Therefore, CDSCs exhibit greater potential as seed cells for repairing the BM niche.

Aplastic anemia represents a clinically challenging BM failure syndrome characterized by pancytopenia and marrow hypoplasia, which arises from both HSC depletion and microenvironmental dysfunction.^[^ ^39^ ^]^ Current treatment options, including HSCT and immunosuppressive therapies, are limited by patient age, physical condition, and potential side effects.^[^ ^40^, ^41^ ^]^ Although CsA is often combined with anti‐thymocyte globulin (ATG) for aplastic anemia treatment, CsA monotherapy exhibits limited efficacy and is associated with significant adverse effects.^[^ ^42^ ^]^ BMSCs derived from aplastic anemia patients are severely impaired in their function.^[^ ^43^ ^]^ CDSCs can be readily cultured and expanded in vitro while expressing BMSC markers, making them a promising alternative for therapeutic applications. While cultured CDSCs alone only modestly prolonged survival in aplastic anemia mice, their combination with CsA significantly improved outcomes compared to CsA monotherapy. This combination enhanced BM cell counts, reduced apoptosis rates, inhibited infiltration of CD4^+^ and CD8^+^ T cells in the BM, peripheral blood, and spleen, and accelerated blood cell recovery. Thus, the combination of CDSCs and CsA demonstrates therapeutic potential comparable to other established aplastic anemia treatments.^[^ ^43^, ^44^, ^45^, ^46^ ^]^

In conclusion, our findings position CDSCs as a versatile therapeutic platform for BM niche restoration. By concurrently reconstructing the stromal microenvironment and safeguarding hematopoietic progenitors, CDSCs address several critical limitations inherent in current transplantation strategies. While the molecular mechanisms underlying CDSC differentiation within the BM niche merit further investigation, their facile expansion and multimodal mechanisms of action render them particularly promising for treating radiation injury, aplastic anemia, and potentially other hematologic disorders associated with niche dysfunction.

A schematic diagram illustrating the mechanism by which CDSCs regenerate the damaged BM niche to facilitate hematopoietic reconstitution. BM failure involves damage to both hematopoietic and non‐hematopoietic cells within the BM microenvironment. CDSCs exhibit the potential to differentiate into endothelial cells, vascular pericytes (including Nestin^+^ cells, CAR cells, and Lepr^+^ cells), as well as osteoblasts. These differentiated cells are capable of secreting additional pro‐hematopoietic cytokines. Furthermore, CDSCs can inhibit apoptosis and reduce DNA damage in injured hematopoietic stem and progenitor cells (HSPCs), referred to as rescued HSPCs including RHSCs and RHPCs, while enhancing the hematopoietic function of donor‐derived HSPCs including DHSCs and DHSPCs. In summary, CDSCs effectively restore the functionality of the impaired BM niche.

## 4 Experimental Section

### Mice

All mouse experiments were approved by the Animal Ethics Committee of the Army Medical University (AMUWEC20212144, Chongqing, China). In this study, CByB6F1 and three types of C57BL/6J mice, namely, CD45.1, ZsGreen, and CD45.2, were utilized. The CD45.1 and ZsGreen mice were purchased from Cyagen (Guangzhou, China), whereas the CD45.2 mice were sourced from Vital River Laboratory Animal Technology Co., Ltd. (Beijing, China) and Chongqing Tengxin Biotechnology Co., Ltd. (Chongqing, China). All mice were specifically housed in the laboratory animal center affiliated with the Army Medical University and the Chongqing International Institute for Immunology. They were provided with ad libitum access to appropriate food and water. The housing environment was strictly controlled, maintaining optimal temperature, humidity, and lighting conditions under a 12 h light‐dark cycle.

### Preparation of isoCDSCs and CDSCs

The costal cartilages were obtained from neonatal CD45.1 or ZsGreen C57BL/6J mice within one week after birth. The cartilage tissues were digested in an enzyme solution containing 0.2% type II collagenase (C6885‐1G, Sigma–Aldrich, USA), dissolved in DMEM/F12 medium (SH30023.01, Cytiva, USA), at 37 °C for 20 min with agitation at 90 rpm. Following digestion, the samples were washed with PBS (C10010500BT, Gibco, USA) to remove surrounding muscle tissue. Subsequently, a second enzymatic digestion was performed under the same conditions for 35 min at 37 °C and 90 rpm. After digestion, the tissues were pipetted up and down repeatedly to obtain a single‐cell suspension in PBS supplemented with 2% fetal bovine serum (FBS, FBSSR‐01021‐500, Cyagen, China). These isolated cells were designated as isoCDSCs in this study. CD45^−^CD51^+^ cells were further fluorescence‐activated cell sorter (FACS)‐sorted from the isoCDSC population and referred to as CDSCs.

### Irradiation and Transplantation Assay

For all lethal irradiations, mice were subjected to 9.5 Gy irradiation (split‐dose of 5 and 4.5 Gy administered 4 h apart using Co‐60 γ‐rays) one day prior to transplantation. In this isoCDSCs transplantation (isoCDSCs‐T) assay, 5 × 10^5^ isolated CDSCs (CD45.1/ZsGreen) from neonatal mice were transplanted via intraorbital vein injection into lethally irradiated 16 weeks old WT recipient mice (CD45.2). For BMT, 5 × 10^5^ BM cells (CD45.1/ZsGreen) isolated from neonatal or 8–12 weeks old donor mice were transplanted via intraorbital vein injection into lethally irradiated 16 weeks old WT recipient mice (CD45.2). For the transplantation assay involving HSCs or MPPs alone or in combination with purified CDSCs, HSCs (ZsGreen), MPPs (ZsGreen), and CDSCs (CD45.1) were sorted separately. Subsequently, 50 HSCs, 1000 MPPs alone or in combination with 5 × 10^5^ purified CDSCs (CD45^−^CD51^+^) were transplanted via intraorbital vein injection into lethally irradiated 8–12 weeks old WT recipient mice (CD45.2).

For the noncompetitive repopulation assay of isoCDSCs‐T, 5 × 10^5^ rescued BM cells (Rescued‐HSCT/CD45.2) were transplanted via intraorbital vein injection into lethally irradiated 8–12 weeks old CD45.1 recipient mice, while 5 × 10^5^ donor‐derived BM cells (Donor‐HSCT/CD45.1) were transplanted via intraorbital vein injection into lethally irradiated 8–12 weeks old CD45.2 recipient mice.

For the sub‐lethal irradiation and transplantation assay, cultured CDSCs passaged at P2 or P3 were transplanted via bilateral intramedullary injection into sub‐lethally irradiated (5 Gy, Co‐60 γ‐rays) 8–12 weeks old WT recipient mice (CD45.2). For the single irradiation tibia assay, the tibias of mice were irradiated at 12 Gy one day prior to transplantation. Purified CDSCs (ZsGreen) were then transplanted via intramedullary injection into the irradiated tibias of 8–12 weeks old WT recipient mice (CD45.2).

### Cells Culture

Purified CDSCs were seeded into a 6‐cm cell culture dish containing culture medium composed of minimum essential medium (SH30024.01B, HyClone, USA), supplemented with 10% FBS and 1% penicillin/streptomycin (15 070 063, Thermo Scientific, USA). The cells were cultured at 37 °C in a 5% CO2 incubator (Thermo Scientific, USA). The culture medium was refreshed 2–3 times per week, and the cells were passaged every 5 days. The MSCs used in this study were BM‐derived MSCs obtained from Oricell (MUBMX‐01001, Cyagen, China). These cells were characterized by flow cytometry and demonstrated high expression levels (>70%) of CD29, CD44, and sca‐1. In contrast, they exhibited negligible expression of CD117 and CD31. MSCs were maintained in a specialized culture medium (MUXMX‐90011, Cyagen, China) at 37 °C in a 5% CO_2_ incubator.

### Cells Preparation and Flow Cytometry

All cells used for flow cytometry and obtained from BM, peripheral blood, spleen, and lymph node samples were prepared as described in the previous study.^[^ ^47^ ^]^ The phenotypic analysis of HSPCs and BMSCs was conducted based on classical descriptions.^[^ ^1^, ^48^, ^49^, ^50^ ^]^ The cell surface markers utilized in this study are listed below: c‐Kit (2B8, eBioscience, USA; 2B8, BioLegend, USA), Sca‐1 (D7, eBioscience, USA; D7, BioLegend, USA), Lin (eBioscience, USA; A20, BioLegend, USA), CD150 (TC15‐12F12.2, BioLegend, USA; TC15‐12F12.2, eBioscience, USA), CD48 (HM48‐1, BD, USA), CD45 (30‐F11, BD, USA), CD34 (RAM34, eBioscience, USA), CD16/CD32 (93, eBioscience, USA), CD127 (A7R34, eBioscience, USA), Live/Dead staining reagent (eBioscience, USA), CD45.1 (M1/70, BioLegend, USA; A20, BioLegend, USA), CD4 (RM4‐5, BioLegend, USA), CD8 (53‐6.7, BioLegend, USA), B220 (RA3‐6B2, BioLegend, USA), CD3 (145‐2C11, BD, USA), CD19 (6D5, BioLegend, USA), Gr‐1 (RB6‐8C5, BioLegend, USA), CD115 (AFS98, BioLegend, USA), NK1.1 (PK136, BioLegend, USA), Ter‐119 (Ter119, BioLegend, USA), CD51 (RMV, BioLegend, USA; RMV, eBioscience, USA), γH2A.X (2F3, BioLegend, USA), PDGFRα (APA5, BioLegend, USA), CD31 (390, BioLegend, USA), F4/80 (BM8, BioLegend, USA), CD11b (M1/70, BioLegend, USA), Lepr (AF497, R&D Systems, France), and Apoptosis Detection Kit (88‐8006‐72, eBioscience, USA). Flow cytometry assays and data analysis were performed using LSRFortessaTM X‐20 (BD, USA) and FlowJo v10.0 software (TreeStar, USA).

### CFU Assay

The U‐bottom 96‐well plates were coated with 100 µL of methylcellulose media (M3434, STEMCELL, Canada), supplemented with 1% penicillin/streptomycin, and incubated at 4 °C for subsequent experiments. Subsequently, single HSCs were sorted from the donor‐derived HSCs and rescued HSCs of isoCDSCs‐T recipients into the pre‐coated 96‐well plates and cultured at 37 °C in a 5% CO_2_ incubator. After 14 days of culture, cell colonies were quantified and measured using an inverted fluorescence microscope (Zeiss Axio Observer.3, Zeiss, Germany).

### BMSCs Isolation

This experiment was performed as previously described.^[^ ^49^ ^]^ Briefly, the flushed BM cells, predominantly consisting of hematopoietic cells, were removed from mice with irradiated single tibias. The remaining tibia was then subjected to continuous enzymatic digestion for 30 min at 110 rpm. The enzyme solution consisted of 1 mg mL^−1^ type IV collagenase (17 104 019, Sigma–Aldrich, USA) and 0.5 mg mL^−1^ Dispase (D4693‐1G, GIBCO, USA), dissolved in DMEM. Following digestion, the cells were harvested through repeated pipetting and rinsing, and subsequently resuspended in PBS buffer containing 2% FBS. Finally, CD45^−^CD31^−^ Ter119^−^ cells (BMSCs) were sorted and depleted from the digested cell suspension using a fluorescence‐activated cell sorter (FACS AriaTM Fusion, BD, USA).

### CFU‐F Assay

The sorted 20 000 BMSCs were seeded into 6‐well plates, and 60 000 BMSCs were seeded into 6‐cm cell culture dishes. The cell culture medium alternately consisted of MesenCult Expansion Kit (05513, STEMCELL, Canada) and essential medium (supplemented with 10% FBS and 1% penicillin/streptomycin), and the medium was refreshed every 5 days. After 10 days of culture, the wells were washed twice with PBS and stained with crystal violet staining solution (C0121, Beyotime, China) for 10 min at room temperature, followed by three additional washes with PBS. Finally, the stained cell colonies were quantified and imaged.

### Hematological Parameter Test

The hematological parameter analysis was performed as described in the previous study^[^ ^51^ ^]^ using an automated animal blood cell analyzer (ProCyte Dx^*^, IDEXX BioResearch, USA).

### RNA Extraction and qRT‐PCR

Total RNA was extracted from the collected cells derived from BM flush, BMSCs, and bone tissues in both the BMT group and the isoCDSCs‐T group using the RNA Fast Extract 200 Mini Kit (220010, Fastagen, China), following the manufacturer's instructions. Subsequently, the extracted RNA was reverse‐transcribed into cDNA using the PrimeScript RT Reagent Kit with gDNA Eraser (RR047A, TAKARA, Japan). The transcription levels of RNA were quantified using TB Green Premix Ex Taq II (RR820A, TAKARA, Japan) on a Real‐Time PCR Detection System (LightCycler 96, Roche, Switzerland). The data were calculated using the 2^−ΔΔCt^ method with normalization to β‐actin. All primers used in this experiment are listed in Table S1 (Supporting Information).

### Immunofluorescence

BM cells were collected from the isoCDSCs transplantation group, the control group, and the healthy control group. The cKit^+^ cells were subsequently isolated from these samples using a cKit positive selection kit (18 757, STEMCELL, Canada). The purified cells were uniformly applied onto glass slides at an appropriate density and air‐dried in a biosafety cabinet. The subsequent immunofluorescence staining procedure was performed in accordance with the protocol used for tissue sections. BM tissues were harvested from euthanized mice and fixed overnight at 4 °C in paraformaldehyde (PFA, P1110, Solarbio, China). Subsequently, the fixed tissues underwent decalcification using an EDTA‐based decalcification solution (BL616B, BioSharp, China) for 3 days. Tissue dehydration was performed by incubation in a 30% sucrose solution for 5 h, followed by embedding in OCT compound (4583, Sakura, Japan) and freezing. Frozen sections (7 µm thick) were prepared using a Leica cryostat (Leica, CM1900). The staining procedure was as follows: sections were equilibrated at room temperature for 1 h, washed three times with PBS (5 min per wash), permeabilized with 0.3% Triton X‐100 (P1081, Solarbio, China) at 37 °C for 10 min, and blocked with 5% donkey serum (BB‐71020, BestBio, China) at room temperature for 1 h. Sections were then incubated with primary antibodies overnight at 4 °C, followed by incubation with secondary antibodies at room temperature for 2 h. Nuclei were stained with DAPI (D9542‐1MG, Sigma–Aldrich, USA). Each incubation step was preceded by three 5‐min washes with PBS. All antibodies used in this study are listed below: CXCL12 (ER1902, HuABIO, China), CD31 (AF3628, R&D Systems, France), Lepr (AF497, R&D Systems, France), Sp7 (ab22552, Abcam, UK), Endomucin (SC‐65495, Santa Cruz Biotechnology, USA), donkey anti‐goat IgG 555 (A21432, Invitrogen, USA), and donkey anti‐rabbit IgG 555 (A21208, Invitrogen, USA).

### Aplastic Anemia Mice Model and Treatment

The mouse model of aplastic anemia was established as described in a previous study.^[^ ^52^ ^]^ Briefly, 8 weeks old CByB6F1 mice (BALB/cBy × C57BL/6J F1) were obtained from Vital River Laboratory Animal Technology Co., Ltd. Within 4–6 h after pre‐irradiation (5 Gy), 5 × 10^6^ lymph node (LN) cells were transplanted into the mice via intraorbital vein injection. For the treatment of aplastic anemia using cultured CDSCs, P2‐P3 passage CDSCs were bilaterally intramedullary injected into aplastic anemia mice on day 0. For the treatment using purified primary CDSCs, purified CDSCs were intraorbitally injected into aplastic anemia mice on day 1. For the combined treatment of aplastic anemia with cultured CDSCs or purified primary CDSCs and cyclosporine A (CsA), or CsA alone, CsA (HY‐B0579, MCE, China) was dissolved in DMSO (ST038, Solarbio, China) and corn oil and administered intraperitoneally to aplastic anemia mice at a dose of 50 µg g^−1^ day^−1^ from days 4–8. All data were collected at 12–16 days post‐treatment based on the survival status of the aplastic anemia mice.

### RNA Sequencing and Analysis

This experiment was divided into three groups: BMT, isoCDSCs‐T, and age‐matched normal mice (Health). At two weeks post‐transplantation, CD45^+^ and CD45^−^ cells were isolated from BM using a CD45‐positive magnetic bead sorting kit (18945, STEMCELL, Canada), and the same amount of both cell types were subjected to RNA‐seq analysis. The resulting datasets were normalized to equivalent sequencing depths, followed by merging the normalized datasets for further analysis. The analyses consisted of two parts: upstream and downstream. For the upstream analysis, raw FASTQ files were assessed for quality control using FastQC and MultiQC. Reads were preprocessed with trimGalore to remove adapters and low‐quality bases, and sequences were aligned to the reference genome using hisat2. The number of reads mapped to each transcript or gene was quantified using featureCounts to generate an expression matrix. For the downstream analysis, the raw count matrix was normalized into a TPM (Transcripts Per Million) matrix. Principal component analysis (PCA) was performed using R software packages factoextra (1.0.7) and FactoMineR (2.9). To evaluate the relative enrichment of gene sets, the ssgsea algorithm was applied to compare sample gene expression data with BM‐related gene sets and calculate enrichment scores. Subsequently, mean enrichment scores were computed for each group. Differential gene expression analysis was conducted using edgeR (3.38.4), followed by Gene Ontology (GO) enrichment analysis using clusterProfiler (4.4.4). Heatmap visualization was generated using Adobe Photoshop (1.0.12). Both raw and processed RNA‐seq data have been deposited in the Genomics Expression Omnibus (GEO) database under accession number GSE289080.

### 10 × Genomics scRNA‐seq and Data Analysis

Primary and cultured CDSCs at passages P2‐P3 were subjected to single‐cell RNA sequencing (scRNA‐seq) using the MobiDrop platform (MobiDrop, China). Briefly, cell viability was assessed by Trypan blue staining (C360I‐2, Beyotime, China) under a microscope. A total of 20 000 viable cells were prepared for library construction on the Chromium Controller (10 × Genomics, USA), utilizing the Chromium Single Cell 3′ v3.1 Library & Gel Bead Kit (1 000 121, 10 × Genomics, USA) to generate oligo (dT)‐primed cDNA libraries. The quality of these libraries was evaluated using an Agilent TapeStation High Sensitivity D5000 ScreenTape system, followed by sequencing on the NovaSeq 6000 platform (Illumina, USA) with a data output of 200 Gb. The scRNA‐seq analysis was primarily conducted based on a previously described method.^[^ ^11^ ^]^ Samples were processed using Cell Ranger v7.0.0 for demultiplexing, barcoding, and single‐cell 3′ gene counting, with cDNA inserts aligned to the mm10/GRCm38 reference genome. Quality control (QC), hypervariable gene identification, dimensionality reduction, and unsupervised clustering were performed using Seurat v4.2.0. Low‐quality cells were excluded based on UMI counts, gene detection numbers, and mitochondrial gene proportions. The analysis pipeline included normalization and log transformation via NormalizeData; identification of the top 2000 variable genes using FindVariableFeatures; scaling of the expression matrix through ScaleData; retention of the top 10 principal components by RunPCA; and visualization of cell embeddings using RunUMAP. Batch effects were mitigated using Canonical Correlation Analysis (CCA) for feature combination and shared structure identification, with results visualized using pheatmap. For trajectory analysis, monocle3 v1.3.4 was employed for object construction, normalization, principal component analysis (PCA), trajectory building, and uniform manifold approximation and projection (UMAP) integration. Final visualizations were generated using Adobe Photoshop v1.0.12 and ClusterGVis v0.1.1. Both raw and processed scRNA‐seq data have been deposited in the GEO database under accession number GSE289526.

### Statistics and Data Availability

All data were analyzed using GraphPad Prism version 10.0. All experiments in this study were independently repeated at least three times. One‐way analysis of variance (ANOVA) or two‐tailed Student's *t*‐tests were performed to analyze the results of multiple groups or pairwise comparisons, respectively. The Kaplan–Meier method was used to analyze the overall survival, and the log‐rank test was used to determine the difference. Data presented as Mean ± SEM. Significance levels are indicated as follows: ^*^ *p* < 0.05, ^**^ *p* < 0.01, ^***^ *p* < 0.001, ^****^ *p* < 0.0001, and ns denotes non‐significant findings.

### Ethical Approval

All mouse experiments were approved by the Animal Ethics Committee of the Army Medical University (AMUWEC20212144), Chongqing, China.

## Conflict of Interest

The authors declare no conflict of interest.

## Author Contributions

R.D., Z.L., P.F., and D.L. contributed equally to this work. Conceptualization, Y.Z., B.N., Y.W., C.Z., and R.D.; methodology, R.D., Z.L., and P.F.; software, X.S., S.W., and R.C.; wet laboratory experiments: R.D., Z.L., and P.F.; assisted by R.Z., W.S., Y.T., L.X., Y.R., and J.W.; data analysis, R.D., Z.L., P.F., and D.L.; writing‐original draft preparation, R.D.; writing‐review and editing, Y.Z., B.N. All authors have read and agreed to the published version of the manuscript.

## Supporting information



Supporting Information

Supplementary Table 1

## Data Availability

RNA‐seq data and scRNA‐seq data have been deposited in the GEO database under accession number GSE289080 and GSE289526.
