# Supplementary material for: A Novel Therapeutic Strategy for Bone Marrow Failure: Niche Rejuvenation Using Costal Cartilage‐Derived Stem Cells
Source: Adv Sci (Weinh). 2025 Aug 27;12(44):e07794. doi: 10.1002/advs.202507794 (PMC12667493; doi:10.1002/advs.202507794)
Supplement: Supplementary file 1 — Supporting Information [file ADVS-12-e07794-s001.docx]

**Supporting information**

**A Novel Therapeutic Strategy for Bone Marrow Failure: Niche Rejuvenation Using Costal Cartilage-Derived Stem Cells**

Rui Dong^1,2,3†^, Zhiguo Ling^2, 3†^, Pengyuan Fan^3,5†^, Debao Li ^3†^, Jinsong Wang^2^, Wenjiong Shi ^3^, Rui Zuo^4^, Runfeng Chen^3^, Xuemin Sun^2^, Lang Xiao^2^, Yushi Ran^2^, Shucheng Huang^3,5^, Yi Tian^2^, Chao Zhang^4^*, Yuzhang Wu^2,3^*, Bing Ni^1^*, Yi Zhang^3,5^*

^1^ Department of Pathophysiology, College of High Altitude Military Medicine, Army Medical University, Chongqing 400038, China

^2^ Institute of Immunology, Army Medical University, Chongqing 400038, China

^3^ Chongqing International Institute for Immunology, Chongqing 401338, China

^4^ Department of Orthopedics, Xinqiao Hospital, Army Medical University, Chongqing 400038, China

^5^ School of Pharmacy and Bioengineering, Chongqing University of Technology, Chongqing 400054, China

*Correspondence:

tmmuzc@tmmu.edu.cn (C.Z.), wuyuzhang@iiicq.vip (Y.W.), nibing@tmmu.edu.cn (B.N.), zhangyi@iiicq.vip (Y.Z.)

†These authors have contributed equally to this work.

**1. Supplementary Figures and Figure Legends**


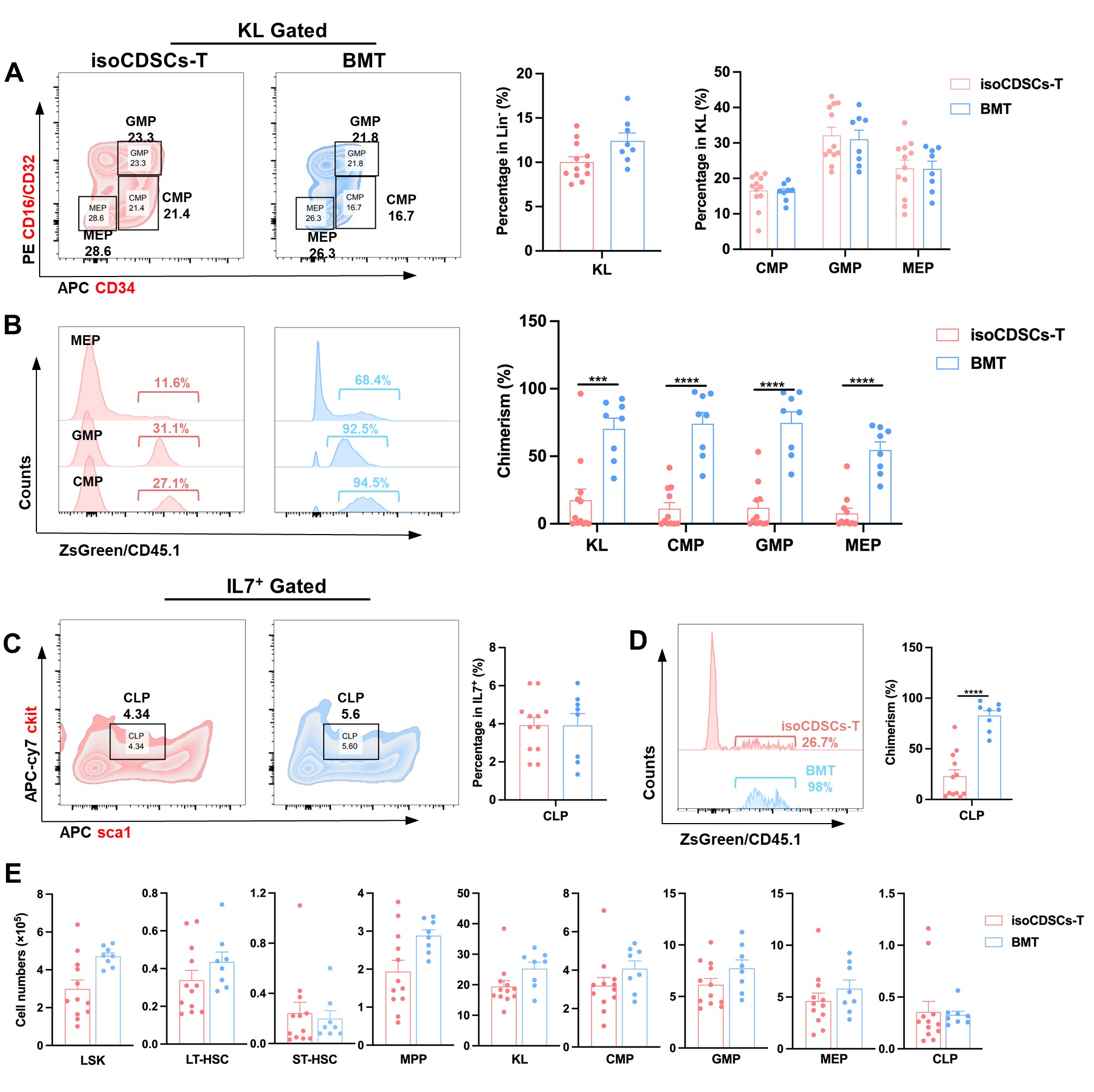


**Figure S1. IsoCDSCs-T achieve a comparable recovery of HSPCs in BM to that achieved by BMT.**

A-D. Percentage and donor chimerism of HPCs and CLPs in BM at 16 weeks post-transplantation for the isoCDSCs-T group (n = 12), and the BMT group (n = 8).

E. Number of HSPCs in the BM (harvested from bilateral femurs and tibias) of recipient mice at 16 weeks post-transplantation for the isoCDSCs-T group (n = 12), and the BMT group (n = 8).

Data presented as Mean ± SEM. ****P* < 0.001, *****P* < 0.0001. P values were calculated by the two-tailed Student’s *t*-test.


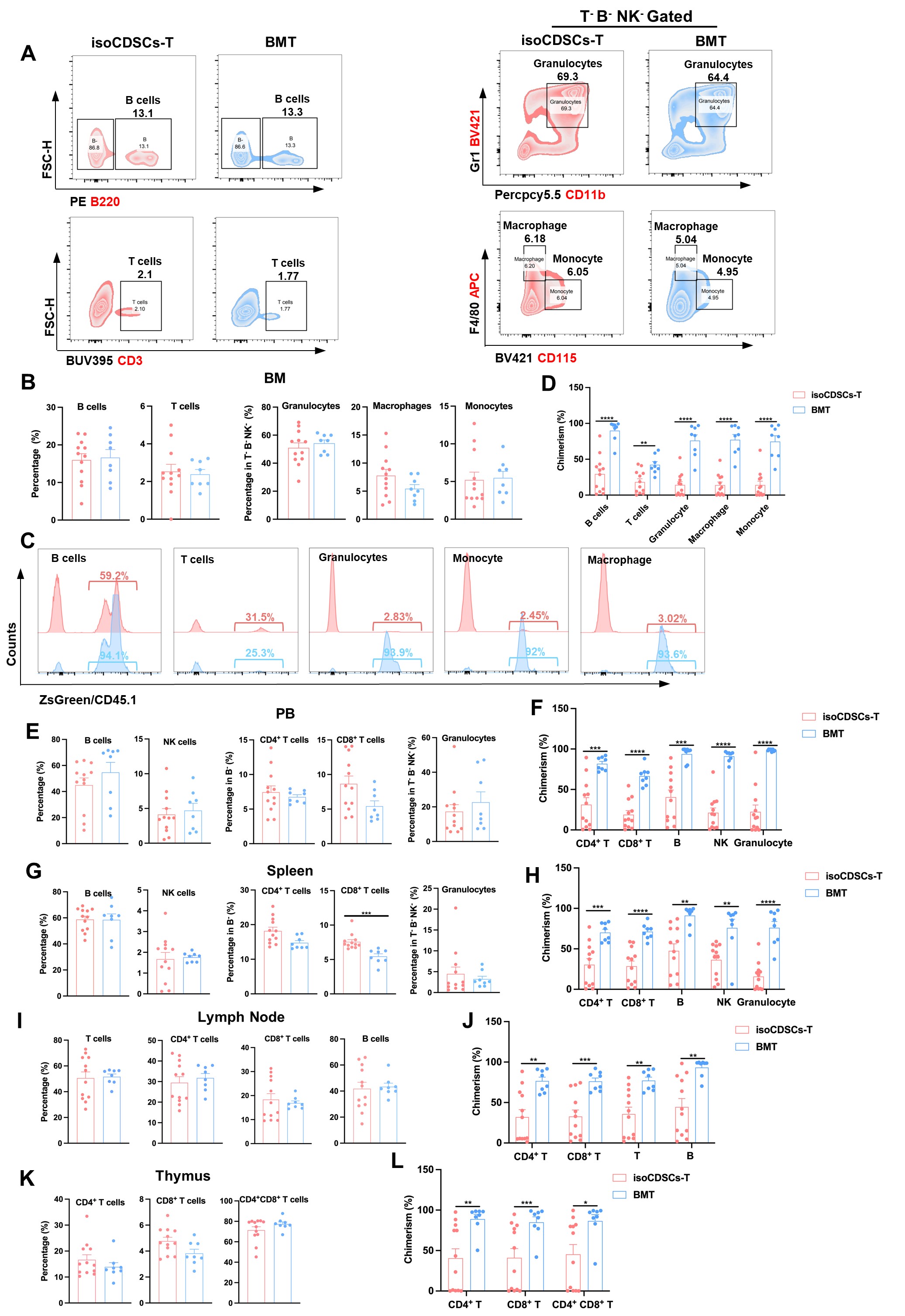


**Figure S2. IsoCDSCs-T achieve a comparable recovery of mature immune cells to that achieved by BMT.**

A-L. Percentage and donor chimerism of mature immune cells in the BM, peripheral blood (PB), spleen, lymph nodes, and thymus of isoCDSCs-T and BMT recipient mice at 16 weeks post-transplantation.

Data presented as Mean ± SEM. **P* < 0.05, ***P* < 0.01, ****P* < 0.001, *****P* < 0.0001. P values were calculated by the two-tailed Student’s *t*-test.


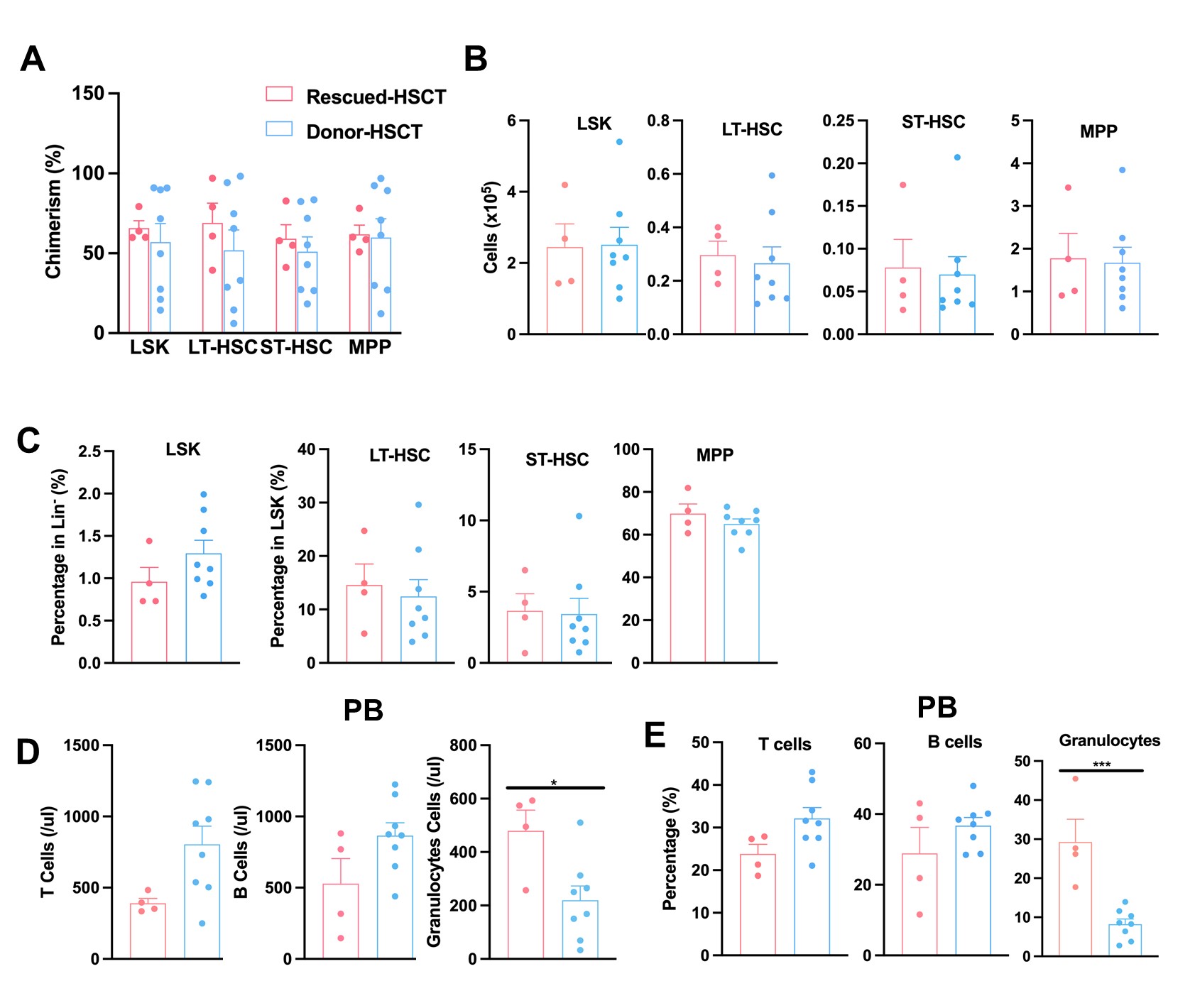


**Figure S3. Rescued irradiated HSCs exhibit robust long-term hematopoietic reconstitution capability.**

A-E. Donor chimerism and quantity of HSPCs in BM (harvested from bilateral femurs and tibias) and quantity of immune cells in PB of non-competitive secondary recipient mice at 16 weeks post-transplantation (Rescued-HSCT, n=4; Donor-HSCT, n=8).

Data presented as Mean ± SEM. **P* < 0.05, ****P* < 0.001. P values were calculated by the two-tailed Student’s *t*-test.


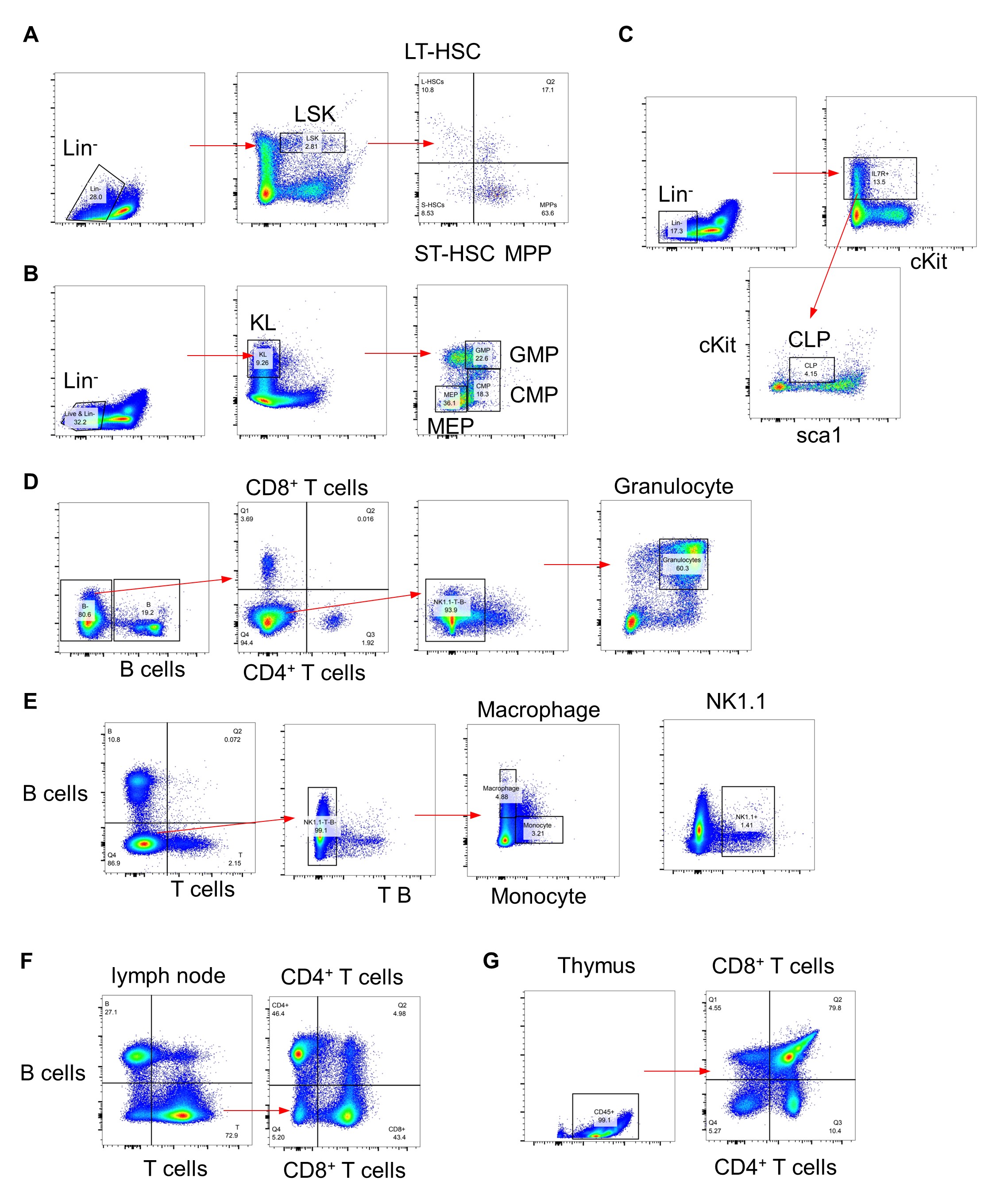


**Figure S4. Flow cytometry plots showing the distribution of HSPCs and mature immune cells as related to Figure 2.**

A-E. Flow cytometry plots showing the distribution of HSPCs (LT-HSCs, ST-HSCs, MPPs), HPCs (CMPs, GMPs, MEPs), CLPs, and mature immune cells (granulocytes, B cells, T cells, macrophages, monocytes, NK cells) in the BM.

F. Flow cytometry plots illustrating the distribution of B cells, T cells, CD4^+^ T cells, and CD8^+^ T cells in the lymph nodes.

G. Flow cytometry plots illustrating the distribution of CD4^+^ T cells, CD8^+^ T cells, and CD4^+^ CD8^+^ T cells in the thymus.

**
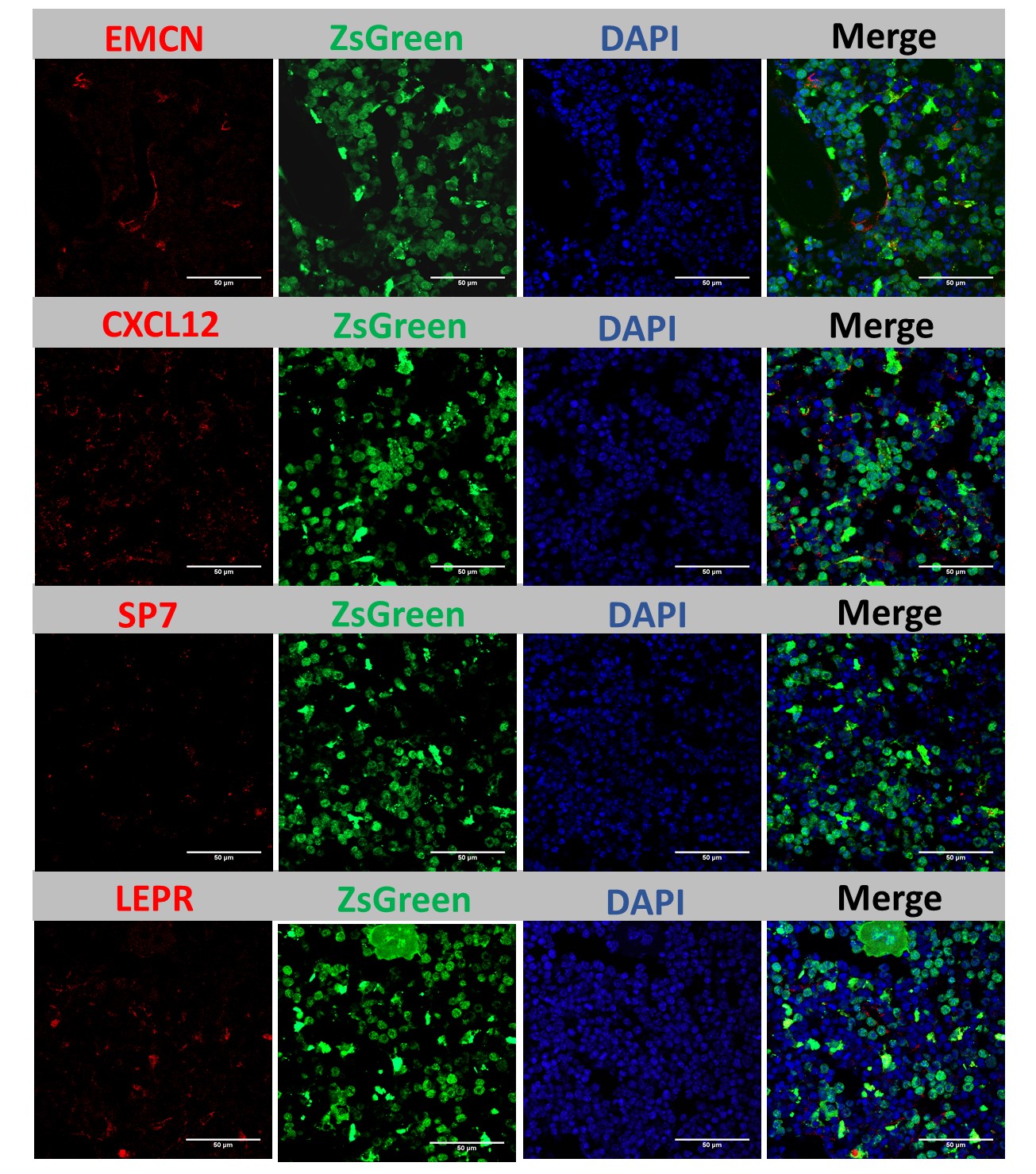
**

**Figure S5. Immunofluorescence imaging showing the expression of BMSCs markers in BMT.**

Immunofluorescence imaging of BM tissues at 8 weeks post-transplantation in BMT recipient mice.

**
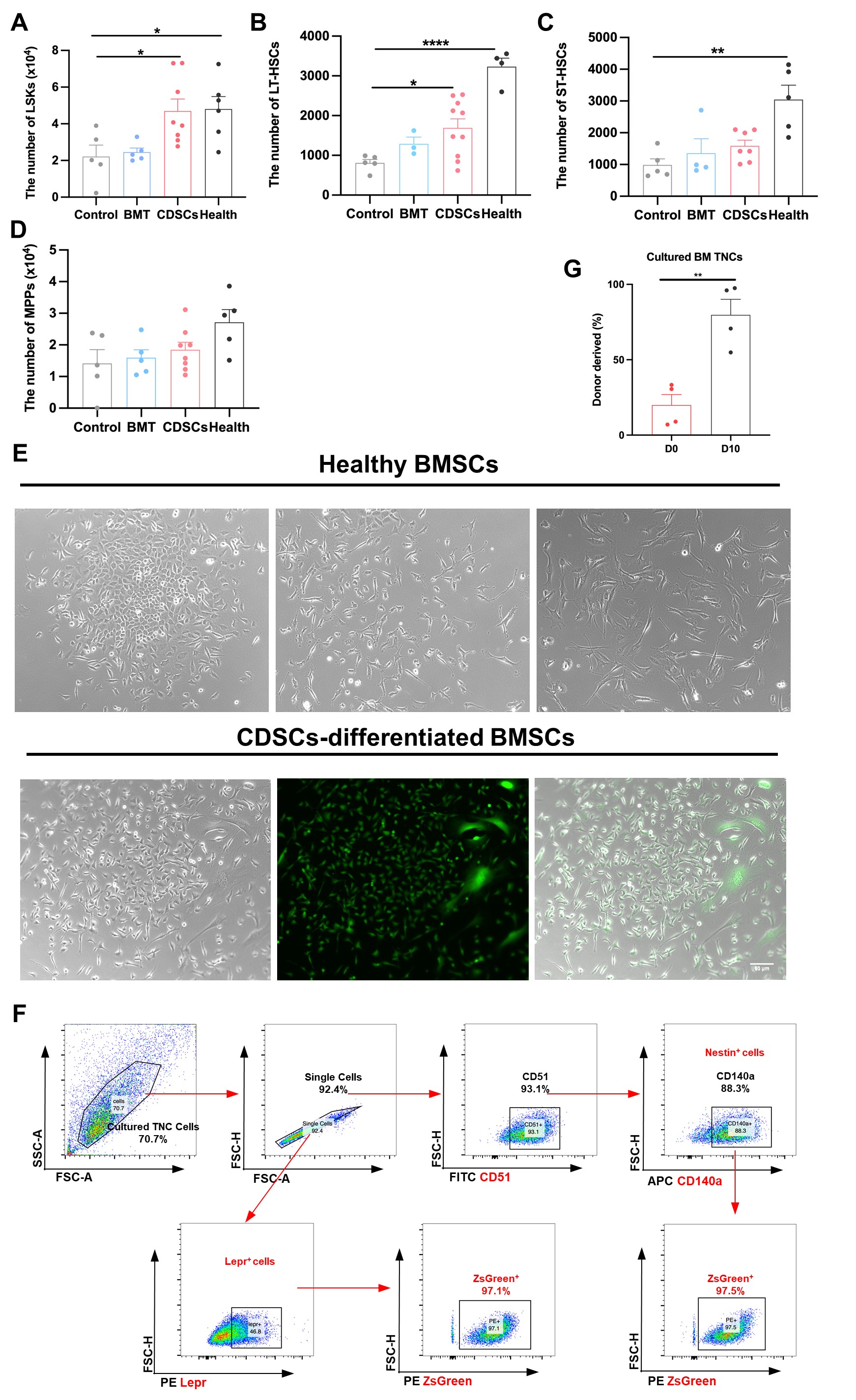
**

**Figure S6. CDSCs-differentiated BMSCs exhibit enhanced proliferative capacity and maintain a healthy morphology.**

A-D. Quantification of BM HSPCs (LSKs, LT-HSCs ST-HSCs, MPPs, harvested from unilateral tibias) in CDSCs, BM transplantation (2-3 weeks post-transplantation), control and health groups.

E. The morphology of BMSCs *in vitro* derived from irradiated single tibia, transplanted recipient mice at 3 weeks post-transplantation, and healthy BMSCs.

F. Flow cytometry plots for the expressed BMSC markers of CDSCs differentiated BMSCs after 10 days of *in vitro* culture.

G. Comparison of chimerism of BMSCs (derived from irradiated tibia and cultured CDSCs transplanted recipient mice at 3 weeks post-transplantation) before and after 10 days *in vitro* culture.

Data presented as Mean ± SEM. **P* < 0.05, ***P* < 0.01, *****P* < 0.0001. P values were calculated by the one-way ANOVA test or the two-tailed Student’s *t*-test.


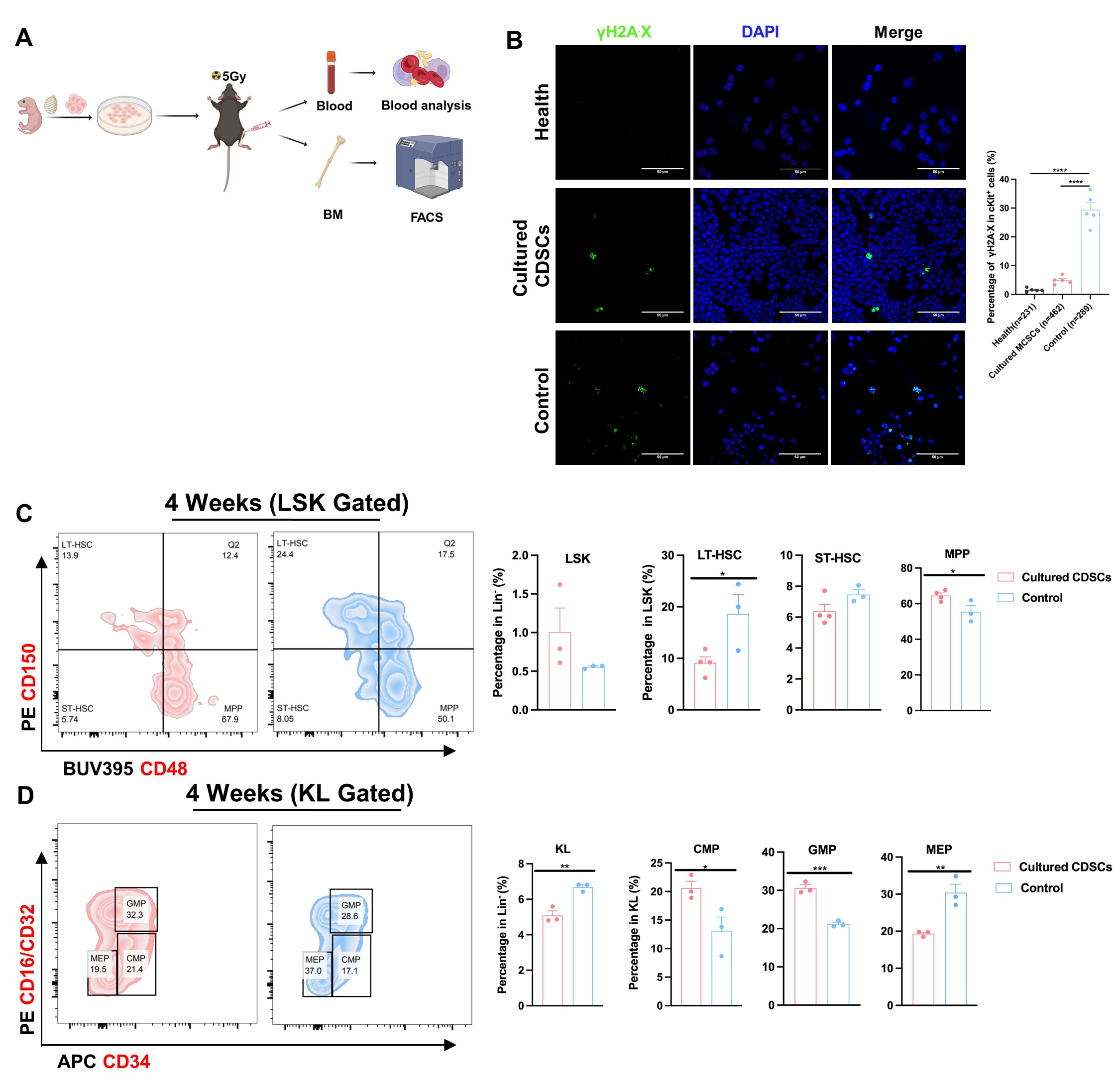


**Figure S7. Cultured CDSCs express suppress the senescence of LT-HSCs.**

A. Summary of the transplantation of cultured CDSCs into sub-lethally irradiated mice via bilateral intramedullary injection.

B. Immunofluorescence imaging of cKit^+^ cells at 3 weeks post-transplantation with cultured CDSCs (cKit^+^ cell numbers, n=462), Health (cKit^+^ cell numbers, n=231) and Control (cKit^+^ cell numbers, n=289) group.

C and D. Percentage of HSPCs and HPCs at 4 weeks post-transplantation with cultured CDSCs (n = 4) compared to the control group (n = 3).

Data presented as Mean ± SEM. **P* < 0.05, ***P* < 0.01, ****P* < 0.001, *****P* < 0.0001. P values were calculated by the one-way ANOVA test or the two-tailed Student’s *t*-test.


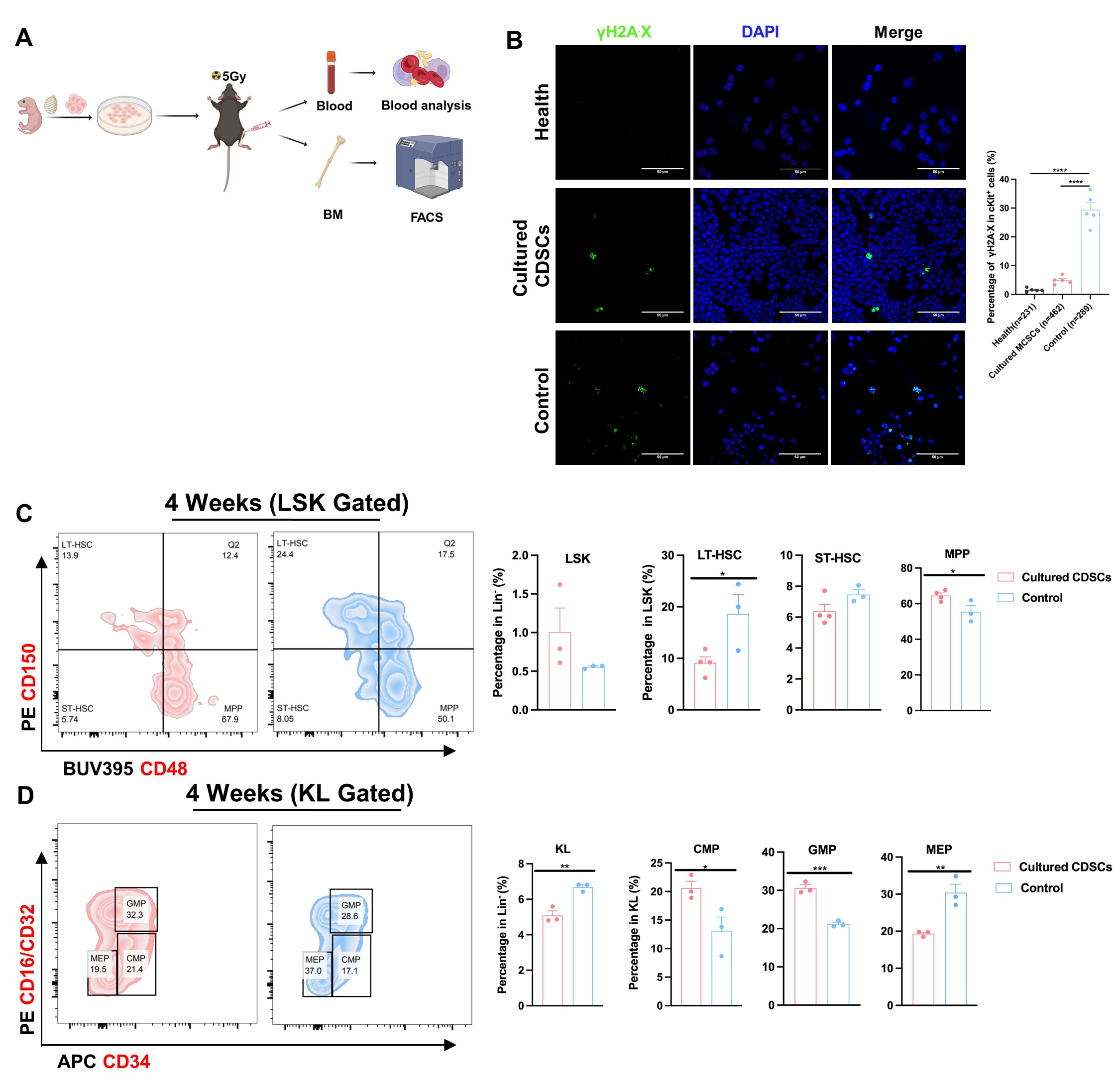


**Figure S8. Cultured CDSCs express BMSC markers.**

A. scRNA-seq analysis reveals the expression of markers associated with BM mesenchymal cells (Pdgfra and Prrx1), smooth muscle/myofibroblasts (Acta2), arterial endothelial cells, other cell types (Ly6a/Sca1), osteoblasts (Col1a1), Ng2^+^ MSCs (Spp1), Cxcl12-abundant reticular (CAR) cells (Cxcl12 and Kitl/Scf), vascular endothelial cells (Cdh5 and Vcam1), and chondrocytes (SOX9 and Acan).

B. Flow cytometry plots showing BMSC markers’ expression in primary CDSCs.

C. Flow cytometry plots showing BMSC marker expression in cultured CDSCs.

D. Expression levels of BMSC markers in cultured CDSCs at passages 0-3 and 6.


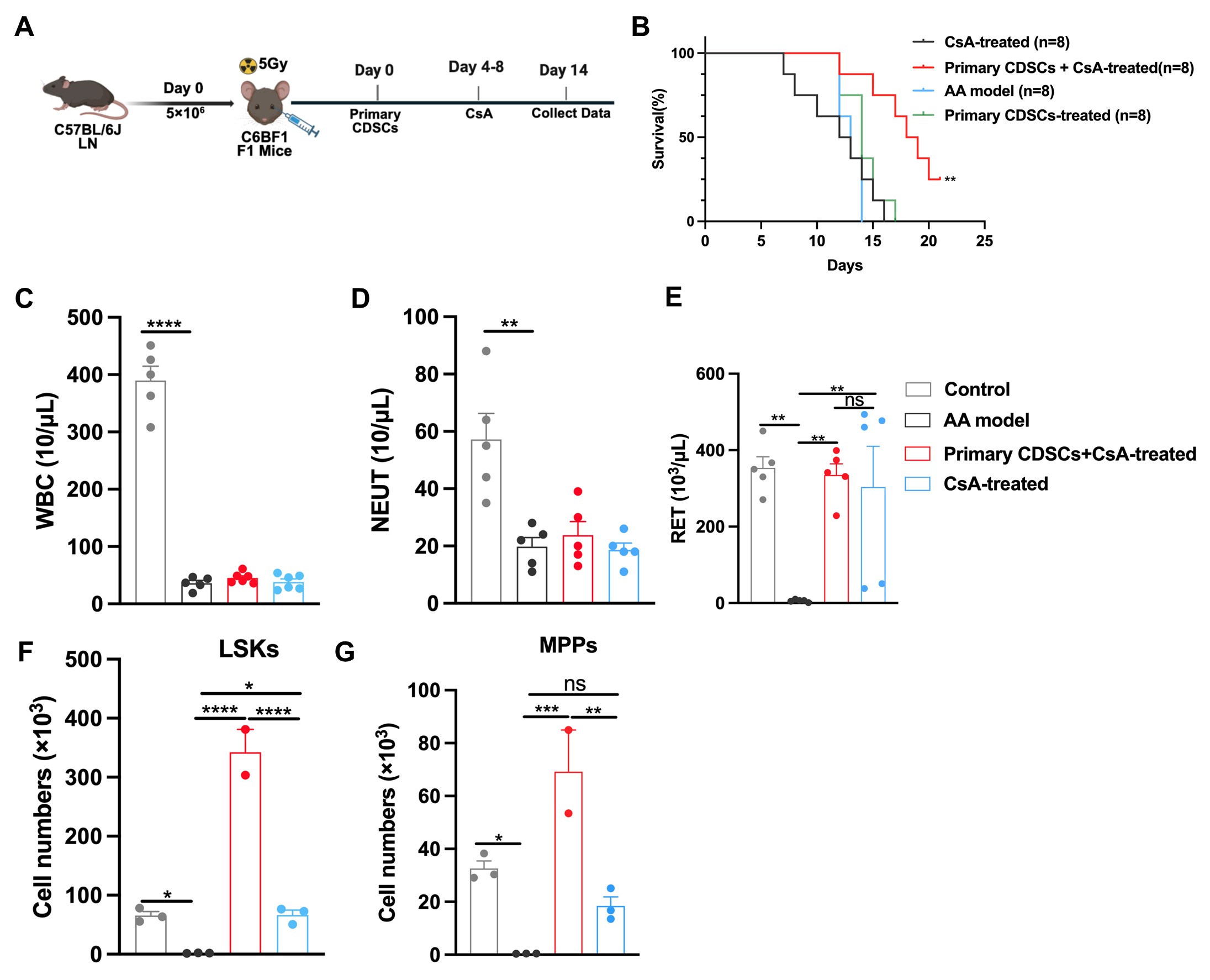


**Figure S9. Primary CDSCs enhance the recovery of BM HSPCs in aplastic anemia mice.**

A. Summary of the treatment for the aplastic anemia (AA) mouse model using purified primary CDSCs and CsA.

B. Survival rates in the AA model, control, CsA-treated, and Primary CDSCs + CsA-treated groups.

C-E. Hematological parameters (WBC, neutrophils, reticulocytes) in the AA model, control, CsA-treated, and Primary CDSCs + CsA-treated groups at 12 days post-treatment.

F and G. Numbers of BM LSKs and MPPs (harvested from bilateral femurs) in the AA model, control, CsA-treated, and Primary CDSCs + CsA-treated groups at 12-16 days post-treatment.

Data presented as Mean ± SEM. **P* < 0.05, ***P* < 0.01, ****P* < 0.001, *****P* < 0.0001, ns, not significant. P values were calculated by the Log-rank test or the one-way ANOVA test.

**2. Supplementary Tables and Table Legends**

**Table S1.** Primers used for RT-qPCR in this study

| **Genes** | **Species** | **Sequence 5'-3'** | |
| --- | --- | --- | --- |
| **Cxcl12** | mouse | F | CCAGAGCCAACGTCAAGCAT |
|  |  | R | CAGCCGTGCAACAATCTGAA |
| **Kitl** | mouse | F | AGGCAGTTAGGTGTAGTTGGGT |
|  |  | R | GTGGCATAAGGGCTCACTCC |
| **Vcam1** | mouse | F | TGAACTGATTATCCAAGTCTCTCCA |
|  |  | R | TGGTGTACGAGCCATCCACA |
| **Spp1** | mouse | F | TCCCTCGATGTCATCCCTGTTG |
|  |  | R | GGCACTCTCCTGGCTCTCTTTG |
| **Adipoq** | mouse | F | TGTTCCTCTTAATCCTGCCCA |
|  |  | R | CCAACCTGCACAAGTTCCCTT |
| **Sp7** | mouse | F | TGCTTGAGGAAGAAGCTCACTAT |
|  |  | R | CCATTGGTGCTTGAGAAGGG |
| **β-actin** | mouse | F | GGCTGTATTCCCCTCCATCG |
|  |  | R | CCAGTTGGTAACAATGCCATGT |

**Table S2.** Top 800 genes of various BMSCs, osteoblast and vascular endothelial cells (see another independently submitted Zip. File).
